# Efficacy and safety of the traditional Chinese formula Shengjiang powder combined with conventional therapy in the treatment of diabetic kidney disease: a systematic review and meta-analysis

**DOI:** 10.3389/fendo.2024.1400939

**Published:** 2024-07-23

**Authors:** Zhuofan Zhang, Yingna Liu, Jiawen Qian, Wenli Jiang, Liqun Cao, Zhiyu Li, Hongbo Chen, Shan Liu

**Affiliations:** ^1^ Center of Clinical Evaluation, The First Affiliated Hospital of Zhejiang Chinese Medical University (Zhejiang Provincial Hospital of Chinese Medicine), Hangzhou, China; ^2^ The First School of Clinical Medicine, Zhejiang Chinese Medical University, Hangzhou, China; ^3^ Department of Nephrology, The First Affiliated Hospital of Zhejiang Chinese Medical University (Zhejiang Provincial Hospital of Chinese Medicine), Hangzhou, China; ^4^ School of Public Health, Zhejiang Chinese Medical University, Hangzhou, China

**Keywords:** Shenjiang powder, heat stagnation, diabetic kidney disease, diabetic nephropathy, meta-analysis

## Abstract

**Objective:**

To investigate the efficacy and safety of Shengjiang powder as a treatment for DKD.

**Methods:**

A comprehensive search was performed in eight databases from their inception to December 30, 2023, to identify relevant RCTs. The inclusion criteria were diagnosis of DKD and intervention including TCM that contained Shengjiang powder. Two researchers independently conducted literature screening and data extraction, utilizing the Rob2 tool and GRADE to assess the quality of the RCTs. Meta-analysis was carried out using RevMan 5.4.1 and Stata 15.0.

**Results:**

As a result of the search, 23 RCTs comprising 1,682 patients. The interventions resulted in significant reductions in all the assessed indicators: 24-h urinary protein, UAER, mALB, BUN, Scr, FBG, 2hPG, HbA1c, total cholesterol, and Triglycerides. Together the results showed that Shengjiang powder, in conjunction with conventional therapy, is an effective treatment of DKD. Subgroup analyses, considering duration, stage, blood glucose control levels, baseline blood glucose levels, and baseline Scr levels indicated that shorter duration treatment had a greater effect on UAER, 2hPG, and HbA1c. Additionally, Shengjiang powder was more effective in reducing 24-h urinary protein, Scr, and 2hPG in stage IV patients compared to corresponding values at other stages. However, with respect to FBG, the treatment was more effective in stage II/III. Shengjiang powder also, reduced Scr levels significantly in patients with higher baseline Scr and reduced urinary protein excretion with stricter blood glucose control. The interventions had additional lipid-regulating effects in cases with looser blood glucose control and led to a remarkable reduction in BUN and Scr levels in patients with FBG > 11.1 mmol/L.

**Conclusion:**

Shengjiang powder may supplement conventional therapy, thus benefiting DKD patients in terms of reducing urinary protein, stabilizing kidney function, and improving blood glucose and lipid metabolism. Considering the significant heterogeneity among studies and limited quality of some reports, our conclusions need to be further verified through analyses utilizing larger, multi-center samples of higher quality.

**Systematic Review Registration:**

https://www.crd.york.ac.uk/prospero/, identifier CRD42024490795.

## 1 Introduction

Diabetic kidney disease (DKD) is a chronic kidney disease characterized by structural injury and dysfunction of the kidney resulting from chronic hyperglycemia. Typical clinical features include progressively increasing levels of urinary protein and deteriorating kidney function (1, 2). In recent decades, the global prevalence of adult diabetes mellitus (DM) and impaired glucose tolerance has significantly increased, with approximately thirty to forty percent of DM patients experiencing various degrees of kidney impairment (3–5). DKD has now emerged as a major public health concern worldwide (6) and is a primary cause of end-stage renal disease (ESRD) (7–9), accounting for over one-third of ESRD cases in Europe and the Americas (10). In China, approximately 30% of type 1 DM and 20% of type 2 DM develop into DKD cases, of which 53% are fatal (11).

The etiology and pathogenesis of DKD remain unelucidated. Current research shows that, other than age and genetic factors, abnormal metabolism of blood glucose and lipids (12), inflammatory responses, and oxidative stress (13) play a role in the pathogenesis of DKD. In addition, abnormal hemodynamics and micro-circulation disturbance of the kidney are closely related to DKD (14, 15).

Currently, clinicians adopt a comprehensive treatment for DKD, emphasizing early intervention. Aside from the basic treatment involving education and diet management, proactive intervention is valued for risk factors, such as hyperglycemia, hypertension, and hyperlipidemia to prevent further kidney damage or cardiovascular disease. With respect to ESRD, patients are treated with hemodialysis and, if necessary, a kidney transplant (16). Additionally, effective treatment also involves strictly controlling blood glucose and blood pressure, as well as blockading the renin-angiotensin-aldosterone system (RAAS) through the use of Angiotensin-converting enzyme inhibitors or Angiotensin receptor blockers (ACEI/ARBs). However, this approach slows the progression of the disease (17) but does not prevent disease onset and eventual progression. With the exception of ACEI/ARBs, for which there is strong evidence of improving the long-term prognosis (18–20), there is little evidence of effective drugs to treat DKD. Thus, it is essential to explore new treatment approaches.

In recent years, more patients have considered traditional Chinese medicine (TCM) as a supplementary treatment for kidney diseases. In a study based in Taiwan, the use of TCM in patients with chronic kidney disease significantly reduced (by ca. 60%) the risk of developing ESRD (21). Furthermore, a meta-analysis of patients with early-stage DKD revealed that the treatment combined with TCM could effectively control disease progression and resulted in fewer side effects (22). The authors of another systematic review also found that the combination of TCM and ACEI/ARBs was superior to ACEI/ARBs alone in reducing the incidence of ESRD and decreasing the levels of 24-h urinary protein, Scr (Serum Creatinine), and BUN (Blood Urea Nitrogen) (23).

From a TCM perspective, DKD, considered a “heat stagnation” pattern, falls in the category of shenxiao derived from long-term xiaoke. The pathogenesis of this condition is essential empty and out solid. Pathological products, such as blood stasis, phlegm obstruction, dampness, and toxic turbidity are outer phenomenons (24, 25). Collaterals blockade is associated with the progression of DKD (26), during which the pathological products and Qi blockade co-cause heat stagnation. Thus, treating DKD requires consideration of the six perspectives: Qi, blood, phlegm, stasis, dampness, and food stagnation. Dissipating them and expelling heat to readjust the Qi movement can effectively ameliorate metabolic disturbances—and this is generally the effect of Shengjiang powder.

Shengjiang powder is a well-known prescription intended to treat “warm” disease. LiShan Yang of the Qing Dynasty called it the general prescription of the “warm” disease that has heat stagnation lurking inside. With the aim of treating “the heat among exterior-interior and triple energizers”, Yang suggested using Shengjiang powder to clear heat, resolve stagnation, and unclog the collaterals (27). Shengjiang powder’s Chinese characters literally mean “to rise and fall” and is essentially a summary of the effectiveness of this formula in TCM, rather than referring to a specific herb like ginger. Its traditional formula consists of the followings: the stiff silkworm, endowed with the Qi of Yangming Dryness-Metal, sends up the lucid yang and dissipates the stagnated turbid phlegm, to avoid further invasion of all stagnated pathogenic factors; cicada slough, regarded as lucid and ethereal to disperse fire, dispels the wind and eliminates the dampness, to detoxify the body; Curcuma longa (rhizome) repels evil by inducing the Qi to untie the sluggish situation and plays the role of diathermy; Rheum officinale (root and rhizome) quells the chaos by dredging throughout the body, and its bitterness determines the function of consolidating yin and draining fire.

Clinically, Shengjiang powder is commonly used to treat externally contracted diseases (28, 29). Yet underlying its application is the principle of “heat stagnation” and resulting pathogenesis, whose symptoms are often manifested as visceral dysfunction. In “Plain Conversation: Major Discussion on the Abstruseness of the Six Kinds of Qi”, it is written that, “the activities of ascent, descent, existing, and entering exist in everything”, “Stoppage of exiting and entering indicates a loss of the life force; failure of Qi to ascend and descend will immediately lead to isolation and loss of Qi.” Obstruction of Qi movement results in the body’s “stagnation”, meaning the inability to regulate the necessary function of raising or lowering. Consequently, whether the disease is contracted externally or internally, it will lead to stagnation and eventually generate heat over time.

Therefore, in the case of biochemical metabolic disorders and hemodynamic abnormalities, it is crucial to determine if DKD cases can be characterized as the “heat stagnation” pattern. If stagnation, turbidity, and heat have amassed in the bodily systems, the Shengjiang powder is recommended to clear and diffuse the stagnant heat reliably. Furthermore, the treatment helps distribute the stretched Qi movement to raise the limpid and lower the turbid.

To date, there have been many randomized controlled trials (RCT) of the use of Shengjiang powder in the treatment of DKD; however, the results of these RCTs cannot be viewed as being highly reliable, due to the small sample size and low test power. In the present study we aimed to evaluate the efficacy and safety, based on RCTs, of Shengjiang powder combined with conventional therapy (CT) in the treatment of DKD.

## 2 Methods

The protocol of our study was registered on 21 January 2024; the registration number in PROSPERO is CRD42024490795. (https://www.crd.york.ac.uk/PROSPERO/) Based on this, we later added additional subgroup analyses.

### 2.1 Search strategy

English language databases (PubMed, Embase, Cochrane Library, International Clinical Trials Registry Platform, Clinical Trials Database, and Grey Literature Database), as well as Chinese databases (CNKI and Wanfang Database) were independently searched by two researchers. The literature retrieval period was from the establishment of the database to December 30, 2023. In addition, we have traced the references included in the studies to supplement the acquisition of relevant research. The search formulation was constructed with a combination of the medical subject heading (MeSH) and free texts. The specific search strategy can be found in **Supplementary Table 1**. Searches were not limited by language or year of publication.

### 2.2 Inclusion and exclusion criteria

In the case of patients diagnosed with DKD, the clinical diagnosis is based on the Expert Consensus on the Prevention and Treatment of DKD (2014 Edition) (30), and the pathological diagnosis is based on Mogensen (31). There were no other restrictions based on gender, age, or region.

The trial group received both CT and TCM, which included all the components of Shengjiang powder (stiff silkworm, cicada slough, Rheum officinale (root and rhizome), and Curcuma longa (rhizome)). The example of administration: one pack daily, 400ml after decocting, divide into twice, and half in the morning and half in the evening. The CT included DM health education, diet management, exercise therapy, and drugs to regulate blood pressure and blood glucose, as well as to optimize blood lipids, etc, regardless of dosage or form. In contrast, the control group received CT only.

The following primary indicators were measured: clinical efficacy (32), 24-h urinary protein, urinary albumin excretion rate (UAER), microalbuminuria (mALB) (16), BUN, and Scr; Secondary indicators were: fasting blood glucose (FBG), 2-h post-load plasma glucose (2hPG), hemoglobin A1c (HbA1c) (33), total cholesterol (TC), and Triglycerides (TG) (34). Safety evaluation index: adverse events.

The study type was limited to RCT. Studies that were excluded were those that contained basic research only, were duplicate publications, and studies in which the main outcomes were not included or the detailed full-text data for statistical analysis was not accessible.

### 2.3 Literature screening

Two researchers first screened the literature independently by reading the title, abstract, and keywords to exclude any literature that did not meet the inclusion criteria. Afterward, they carefully examined all articles and selected only those that met the inclusion criteria for meta-analysis. Lastly, data extraction and cross-checks were performed to ensure the accuracy and completeness of the data. Where there was any disagreement, the team’s third party assisted in deciding whether to include the study or not. The following information was extracted (1): basic study details, namely the study title, first author, journal, and publication date (2); baseline characteristics of the study subjects, such as the sample size, age, gender, and disease stage (3); specific intervention details, including duration (4); key elements of the risk of bias assessment; and (5) outcome indicators, such as UAER and Scr.

### 2.4 Risk of bias and grade of evidence

The risk of bias of included studies was independently evaluated and cross-checked by two researchers according to the Cochrane Handbook’s risk of bias assessment tool for RCTs (35). In the case of any disagreements, they were resolved through discussions or consultations with a third party. The Grading of Recommendation, Assessment, Development, and Evaluation (GRADE) scale was used to assess the certainty of these studies (https://gdt.gradepro.org).

### 2.5 Statistical analysis

The meta-analysis was performed using RevMan5.4.1 software and Stata IC 15.0. Binary data and continuous data were respectively estimated with relative risk (RR) and mean differences (MD), with 95% confidence intervals (CI). The I^2^ test assessed the heterogeneity of the data. The random-effects model is preferred based on the study population, purpose, assumptions and the characteristics of the model (36). When conducting a meta-analysis with less than five studies, a fixed-effect model should be considered instead, as accurately estimating between-study variance or heterogeneity parameters becomes difficult. For heterogeneity sources and the potential relationship between grouping criteria and outcomes, subgroup analyses (37) were separately performed based on the following criteria: the duration of treatment, stage of disease, levels of blood glucose control (HbA1c after treatment), baseline blood glucose levels (pre-treatment FBG) and whether the study provided a specific dose of TCM composition. Additionally, we used Sensitivity analysis to assess the robustness of the results. When ten or more studies were available for comparison, funnel plots, Egger’s test, and the trim-and-fill method were used to detect publication bias. *Post hoc*, we conducted three extra subgroup analyses based on the provision of dose, the difference in diagnostic criteria referred to by each study, and the level of the risk of bias.

## 3 Results

**Figure 1:**
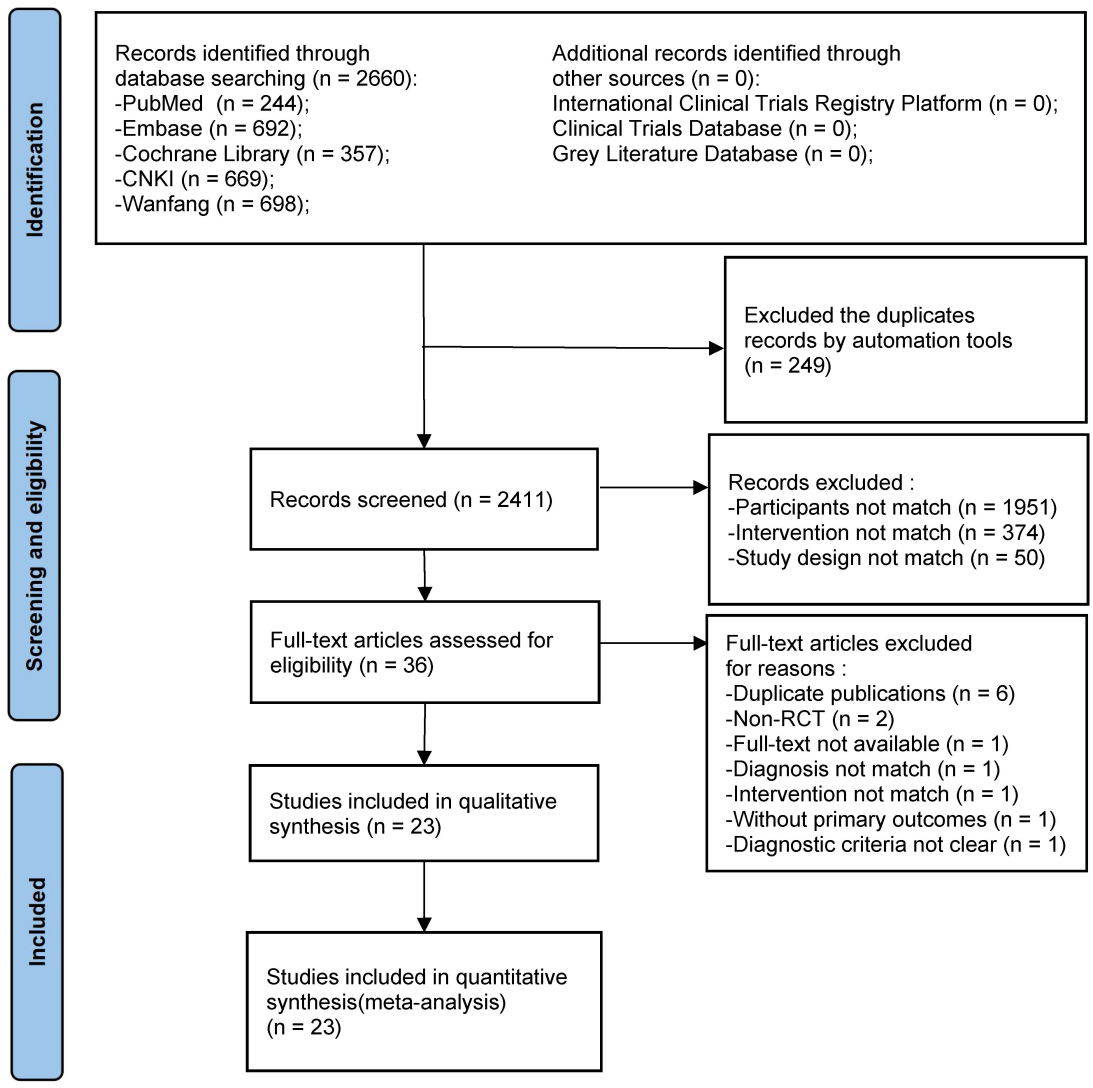
PRISMA flow chart of the systematic review and meta-analysis. Flow chart depicting the number of studies included at each stage of the selection process. RCT, randomized controlled trial.

Initially, we obtained 2,660 relevant articles in the preliminary review of each database. After cascade screening (**Figure 1**), 23 RCTs were selected (38–60).

### 3.1 Characteristics of studies

A total of 1,682 DKD patients participated in the 23 selected studies, in which basic information, namely age, gender, stage of disease, and treatment duration of the enrolled patients were documented. Exceptions were one study that did not report age (60) and one in which gender was not reported (56), as well as three studies in which the course was not specified (52, 58, 60) and one study in which treatment duration was not reported (52).

Disease stage was reported in all but two of 23 the studies (52, 58). Of the 21 studies in which disease stage was given, all but one referred to Mogensen’s stages of DKD. In one study (45) a combination of the CKD stages (G1-G3a) and albuminuria stages (A2-3) was adopted, as recommended by the KDIGO guidelines (61). In two of the studies (55, 56) post-intervention follow-up was carried out, and in another study (56) the post-treatment recurrence rates were reported.

The majority of studies provided specific daily doses of the four-flavor composition of Shengjiang powder, while 5 studies (40, 42, 43, 47, 53) did not mention the doses and one study (49) gave the range of doses. The descriptive statistics analysis results showed that the dose fluctuation from small to large was Curcuma longa (rhizome), Rheum officinale(root and rhizome), Stiff silkworm, and Cicada slough. The average drug use and dosage range of each component are shown in the **Supplementary Table 2**. In terms of diagnostic criteria, one study adopted the diagnostic criteria of KDOQI (62), one was diagnosed according to CSN2021 (16) (refers to KDIGO2020 (61)), and five referred to CDS2014 (30) (refers to ADA2014 (63)). The remaining studies directly used Mogensen’s diagnostic criteria for DKD.

The basic information of the included studies is shown in **Table 1**, and the composition and the specific daily dose of TCM in each intervention is shown in **Supplementary Table 3**.

**Table 1:** Characteristics of included studies.

Studies	Sample	Age(year)	Gender(male,female)	Course(year)	Stage (Mogensen)	Intervention	Comparison	Duration	Outcomes	Diagnostic criteria
	E/C	E/C	E/C	E/C	E/C					
Ma 2024 (44)	40/40	59.0 ± 9.7/55.0 ± 11.0	(19,21)/(15,25)	5.3 ± 2.4/4.9 ± 2.6	III/IV	TCM + CT	Benazepril Hydrochloride Tablets + CT	12 weeks	②③⑤⑥⑦	A
Zhao 2022 (55)	40/40	44.6 ± 3.2/45.3 ± 3.7	(23,17)/(24,16)	9.54 ± 1.75/9.61 ± 1.84(DM)	II/III	TCM + CT	CT	4 weeks	①②③⑤	B
Cheng 2022 (38)	40/40	64.18 ± 6.09/62.00 ± 7.42	(18,22)/(21,19)	11.50 ± 1.56/11.93 ± 1.80(DM) 3.10 ± 0.96/3.30 ± 1.09(DKD)	IV	TCM + CT	CT	12weeks	①②⑤⑥⑨	B
Zhang-2 2022 (54)	46/45	57.88 ± 6.45/58.17 ± 6.05	(27,19)/(24,21)	13.83 ± 1.27/13.49 ± 1.54	III	TCM + CT	CT	8weeks	②③⑤⑥⑦	B
Cao 2022 (45)	30/30	60.06 ± 9.03/57.53 ± 8.64	(20,10)/(15,15)	14.30 ± 6.98/16.17 ± 6.01(DM)	NR	TCM + CT	CT	12weeks	①②④⑦⑧⑨	C
Luo 2021 (51)	38/39	64.21 ± 6.01/62.21 ± 6.60	(20,18)/(21,18)	13.18 ± 3.27/13.31 ± 3.15(DM) 5.92 ± 0.88/5.95 ± 1.05(DKD)	III	TCM + CT	CT	8weeks	①③④⑤⑥⑦⑧⑨	B
Kong 2020 (46)	41/43	52.12 ± 0.55/54.81 ± 10.68	(22,19)/(18,25)	10.22 ± 3.33/9.83 ± 3.34	III	TCM + CT	CT	2months	①③⑦⑧⑨	B
Zhuang-2 2020 (39)	36/36	52.2 ± 7.4/52.6 ± 7.6	(19,17)/16,20)	6.5 ± 1.2/6.4 ± 1.3(DKD)	IV	TCM + CT	CT	8weeks	①②⑤⑥	D
Zhuang-1 2017 (42)	20/20	49.6 ± 7.3/49.8 ± 5.7	(10,10)/(11,9)	8.19 ± 1.02/8.21 ± 1.06	IV	TCM + Tripterygium Glycosides + CT	Benazepril/Telmisartan + CT	8weeks	①②⑦⑨	D
Jiao 2017 (47)	48/48	54.31 ± 14.57/55.47 ± 13.91	(35,13)/(32,16)	8.12 ± 7.31/8.41 ± 7.02	III/IV	TCM + CT	Benazepril + CT	3months	①②④⑤⑥⑦⑧	D
Zhang-1 2016 (41)	42/40	56.4 ± 9.7/55.6 ± 8.9	(24,18)/(22,18)	8.2 ± 4.5/8.1 ± 5.2	III	TCM + CT	CT	24weeks	①②⑦⑨	D
Zheng 2016 (58)	30/30	47.93 ± 15.19/48.33 ± 15.23	(18,12)/(16,14)	NR	NR	TCM + CT	Enalapril maleate + CT	1month	④⑨	D
Bian 2015 (40)	30/30	57.97 ± 9.79/58.30 ± 9.46	(16,14)/(14,16)	7.50 ± 2.73/7.97 ± 2.59	III/IV	TCM + CT	CT	2months	①②⑥	D
Guo 2015 (53)	30/30	52.40 ± 9.05/51.77 ± 9.40	(23,7)/(22,8)	6.8 ± 1.7/7.1 ± 1.5	III	TCM + CT	CT	8weeks	④⑦⑨	D
Chen 2014 (49)	30/30	56.8 ± 2.4/56.2 ± 1.7	(14,16)/(17,13)	7.4 ± 3.1/8.4 ± 2.6(DM)	IV	TCM + CT	CT	12weeks	②⑤⑥⑦⑧⑨	D
Zhou 2014 (43)	30/30	57.47 ± 9.733/57.57 ± 9.846	(16,14)/(15,15)	7.23 ± 2.473/7.40 ± 2.811	III/IV	TCM + CT	CT	12weeks	①②④⑦⑨	D
Gao 2013 (56)	49/49	48.3 ± 4.1/48.4 ± 3.9	NR	4.66 ± 0.32/4.63 ± 0.35	III	TCM + CT	Benazepril Hydrochloride Tablets + CT	4weeks	①③	D
Liu 2012 (57)	40/40	50 ± 5.2/51 ± 6.3	(20,20)/(20,20)	1.8 ± 0.8/2 ± 0.5	IV	TCM + CT	CT	4weeks	①⑤⑥	D
Tang 2011 (59)	38/38	52.3 ± 4.6/50.2 ± 6.8	(20,18)/(21,17)	10.3 ± 6.3/11.2 ± 5.4	IV	TCM + CT	CT	4weeks	①⑤⑥	D
Li-2 2011 (48)	30/30	51.8 ± 8.7/51.4 ± 7.8	(14,16)/(16,14)	9.6 ± 4.4/10.8 ± 4.3	III	TCM + CT	CT	8weeks	②⑦⑧⑨	D
Li-3 2011 (60)	49/41	NR	(54,35)	NR	III/IV	TCM + CT	Benazepril + CT	12weeks	②③⑤⑥⑦	D
Ji 2009 (50)	30/30	57.83 ± 9.68/57.10 ± 9.13	(14,16)/(16,14)	9.30 ± 1.53/9.50 ± 1.80	IV	TCM + CT	CT	8weeks	①②⑤⑦⑧⑨	D
Li-1 2008 (52)	42/34	49.7/48.6	(23,19)/(18,16)	NR	NR	TCM + CT	CT	NR	①②⑤⑥⑦⑧	D

Data are mean ± SD unless otherwise indicated. ① Clinical efficacy; ② 24-h urinary protein; ③ UAER; ④ mALB; ⑤ BUN; ⑥ Scr; ⑦ Blood glucose index (FBG, 2hPG, and HbA1c); ⑧ Blood lipid index (TC and TG); ⑨ Safety indicators and adverse events. A: KDOQI Clinical Practice Guidelines and Clinical Practice Recommendations for Diabetes and Chronic Kidney Disease; B: Chinese Expert Consensus on the Prevention and Treatment of DKD (2014 Edition); C: Chinese Guideline on the Prevention and Treatment of DKD (2021 Edition); D: Mogensen. E, Experimental group; C, Control group; NR, Not reported; DM, Diabetes mellitus; DKD, Diabetic kidney disease; TCM, Traditional Chinese medicine; CT, Conventional therapy; UAER, Urinary Albumin Excretion Rate; mALB, Microalbuminuria; BUN, Blood Urea Nitrogen; Scr, Serum Creatinine; FBG, Fasting Blood Glucose; 2hPG, 2-h post-load Plasma Glucose; HbA1c, Hemoglobin A1c; TC, Total Cholesterol; TG, Triglycerides.

### 3.2 Risk of bias assessment

**Figure 2:**
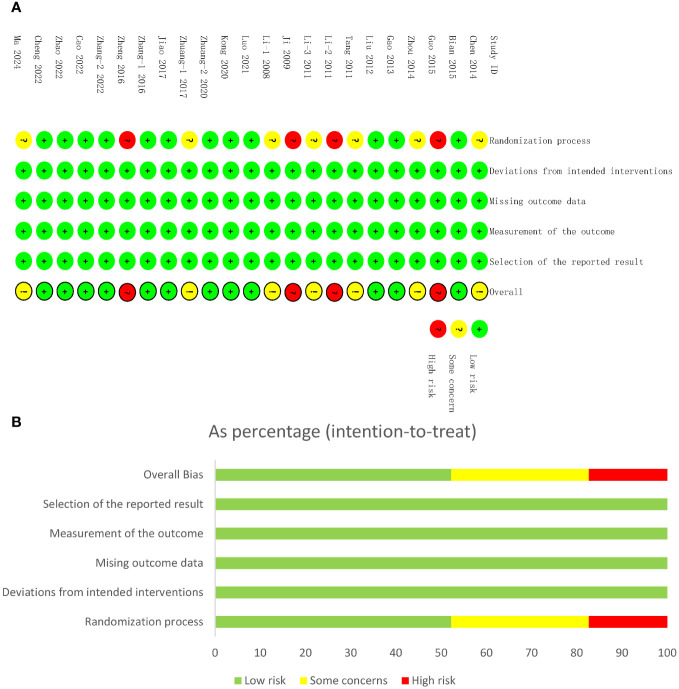
Risk of bias assessments. **(A)** Risk of bias summary of studies included in the meta-analysis. The risk of bias summary. Low risk, unclear risk, and high risk of bias are denoted by ‘+’, ‘?’, and ‘!’, respectively. **(B)** Risk of bias graph. Low risk, unclear risk, and high risk of bias are represented by green, yellow, and red color, respectively.

In terms of randomization methods domain, inappropriate methods were utilized in four of the studies (48, 50, 53, 58), resulting in high risk bias; in another seven studies (42–44, 49, 52, 59, 60), the risk of bias is also a concern, given that specific methods of randomization were not described. In more than half (12 of 23) of the studies, the random number table method was used and in one study (54) the coin toss method was used. None of the studies reported blinding or distributive hiding. In terms of the third domain, four studies (40, 45, 46, 51) reported dropped or excluded cases. No selectivity in reporting results or other bias was found in the included studies (**Figure 2**).

### 3.3 Meta-analysis results of primary outcomes

#### 3.3.1 Clinical efficacy

**Figure 3:**
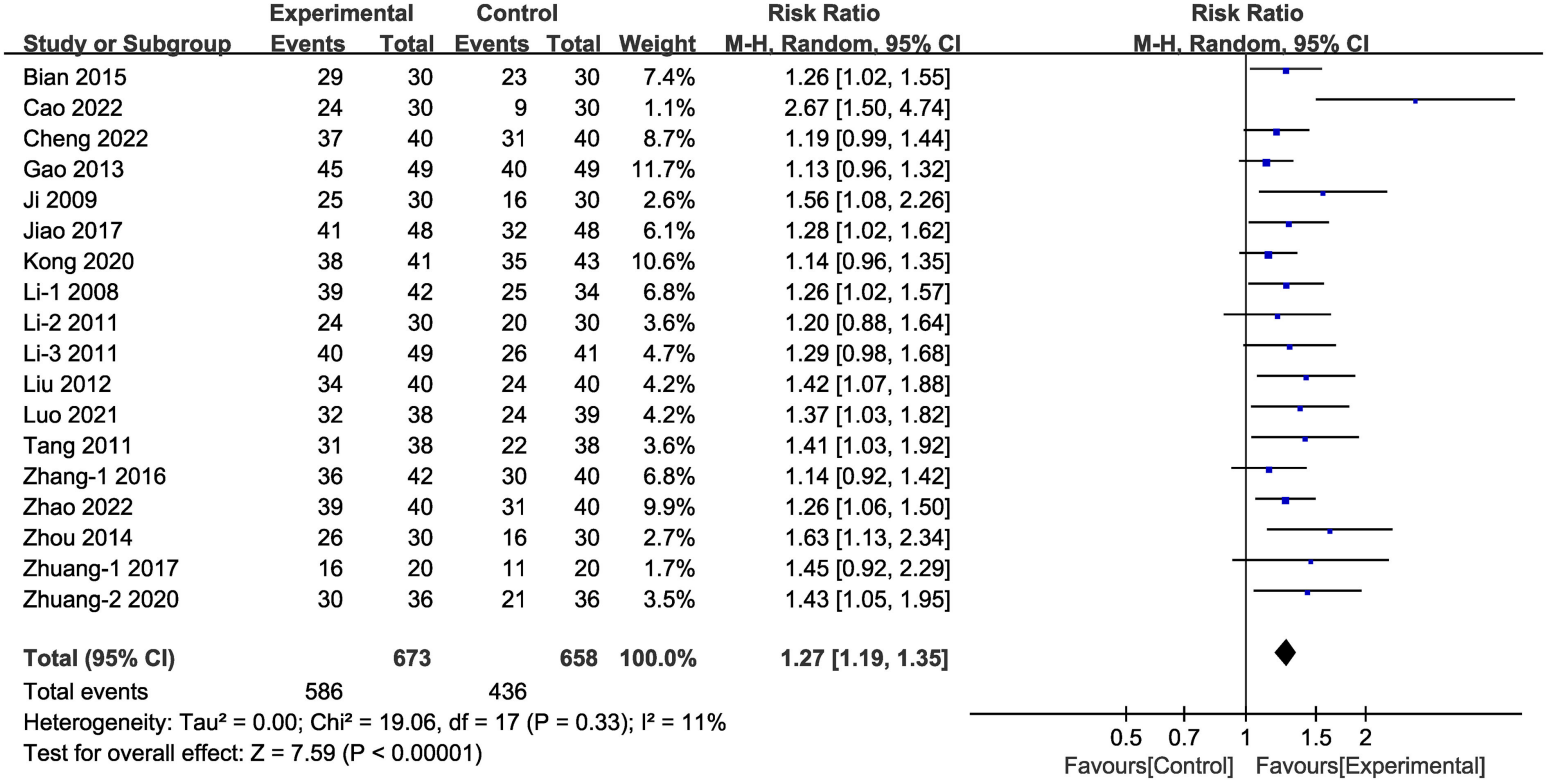
Forest plot illustrating the risk ratio of clinical efficacy with a random effects model.

In the majority (18 of 24) of the selected studies (**Figure 3**), representing 1,331 patients in total, clinical efficacy was reported. Meta-analysis within the random-effects model demonstrated that clinical efficacy was higher in the experimental group than in the control group [RR =1.27, 95% CI (1.19,1.35), P < 0.001].

#### 3.3.2 24-h urinary protein

**Figure 4:**
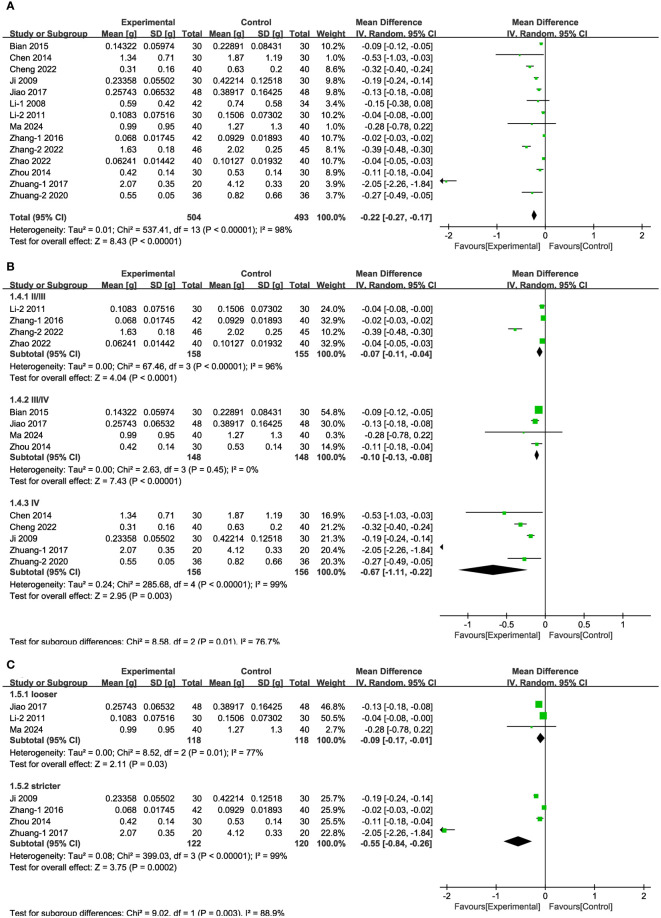
Forest plots illustrating the mean differences of 24-h urinary protein **(A)** overall effect, **(B)** subgroups according to stages, and **(C)** subgroups according to blood glucose control levels, with a random effects model.

In 14 of the studies (**Figure 4A**), representing a total of 997 patients, the level of 24-h urinary protein before and after intervention was reported. Meta-analysis within the random-effects model demonstrated that the interventions could effectively reduce the level of 24-h urinary protein [MD = -0.22, 95% CI (-0.27,-0.17), P < 0.001]. Subgroup analysis was conducted according to duration, stage, blood glucose control levels, and baseline blood glucose levels, and the results revealed that heterogeneity among subgroups still existed. Measures of baseline blood glucose levels and duration indicated that the difference between subgroups’ MDs was not statistically significant (**Supplementary Figure 1**, **Supplementary Table 4**). The beneficial effect of Shengjiang powder combined with CT groups was greater in DKD stage IV patients than in patients with stage II/III disease and was more pronounced in cases with stricter blood glucose control than in looser ones (**Figures 4B, C**).

#### 3.3.3 UAER

**Figure 5:**
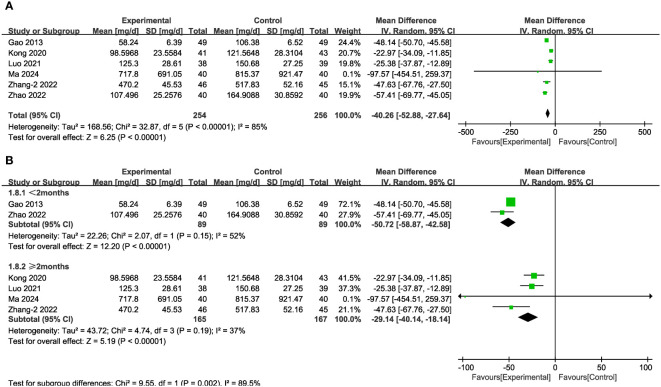
Forest plots illustrating the mean differences of UAER **(A)** overall effect, and **(B)** subgroups according to durations, with a random effects model.

In seven of the studies (**Figure 5A**), representing 510 patients, UAER levels were measured. Meta-analysis within the random-effects model demonstrated that the interventions effectively reduced UAER compared with the corresponding values in the control group [MD = -40.26, 95% CI (-52.88,-27.64), P < 0.001]. Subgroup analysis based on duration revealed that heterogeneity among subgroups decreased. We speculated that the source of heterogeneity is the duration of the interventions. UAER levels were reduced to a greater extent in patients undergoing treatment for less than two months, compared with the corresponding values in cases of longer duration of treatment (**Figure 5B**).

#### 3.3.4 mALB

**Figure 6:**
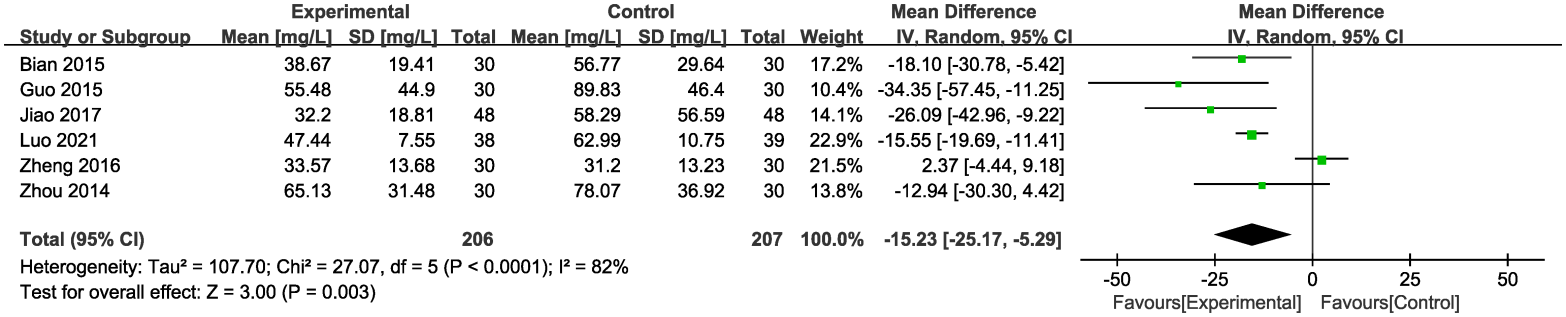
Forest plot of mALB with a random effects model.

In six of the studies (**Figure 6**), representing 413 patients, mALB levels were reported. Meta-analysis within the random-effects model demonstrated that the intervention effectively reduced mALB [MD = -15.23, 95% CI (-25.17,-5.29), P < 0.01].

#### 3.3.5 BUN

**Figure 7:**
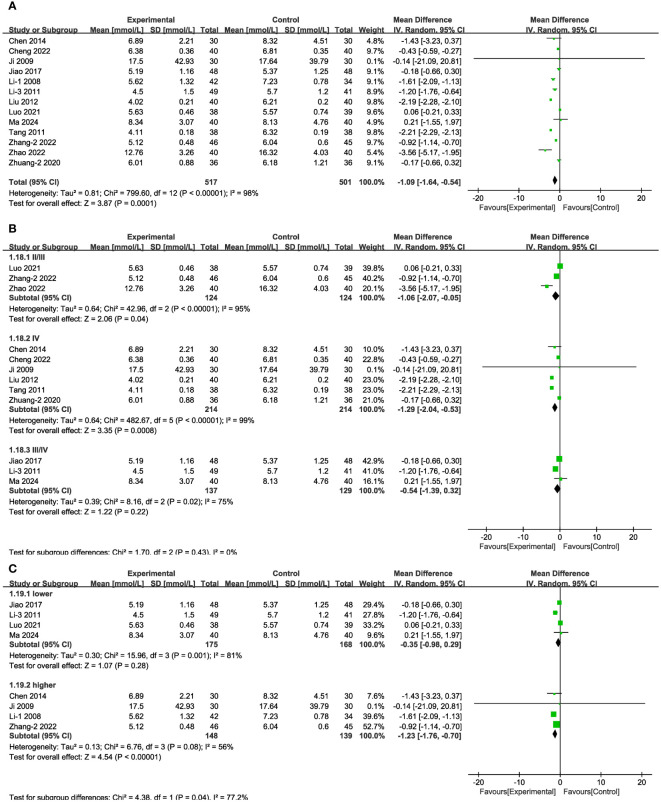
Forest plots illustrating the mean differences of BUN **(A)** overall effect, **(B)** subgroups according to stages, and **(C)** subgroups according to baseline blood glucose levels, with a random effects model.

In 13 of the studies (**Figure 7A**), which together represent 1,018 patients, BUN values were reported. Meta-analysis within the random-effects model showed that, compared with CT alone, the combination of Shengjiang powder with CT resulted in lower BUN values [MD = -1.09, 95% CI (-1.64,-0.54), P < 0.001]. Subgroup analysis results revealed that heterogeneity among subgroups did not decrease, and there was no statistical difference between the MDs of duration subgroups (**Supplementary Figure 1**, **Supplementary Table 4**). As well, there was no statistical difference in BUN values between the stage III/IV group and lower-baseline blood glucose group (FBG < 11.1 mmol/L) (**Figures 7B, C**).

#### 3.3.6 Scr

**Figure 8:**
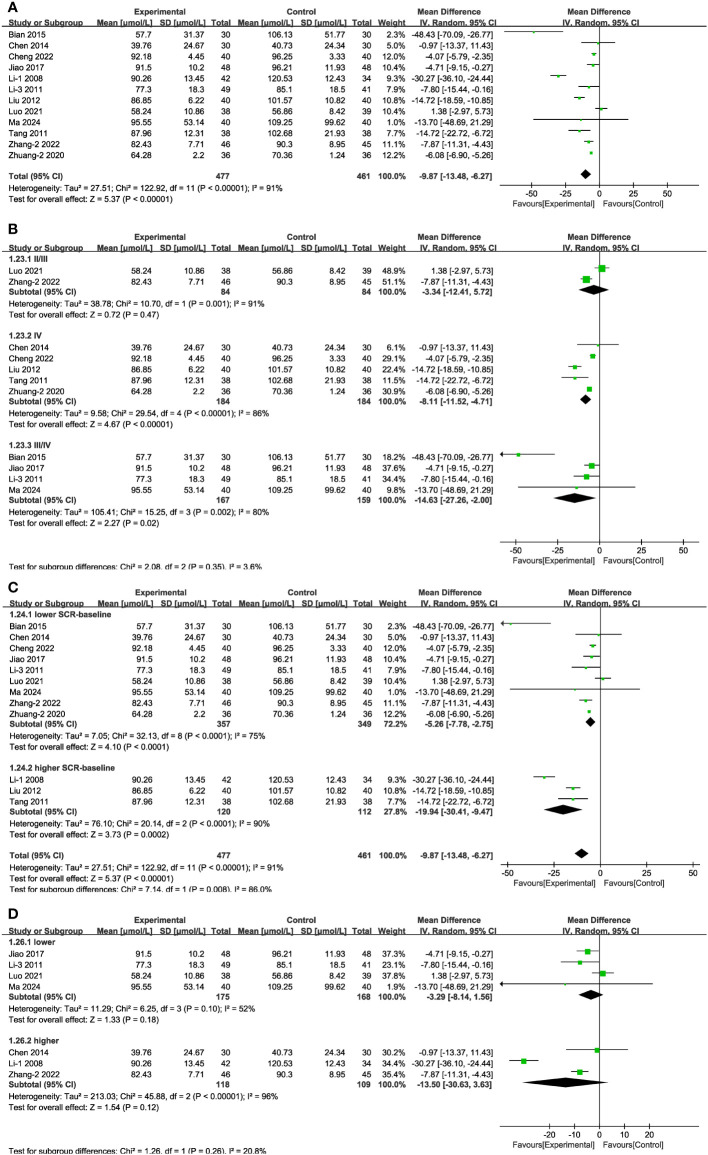
Forest plots illustrating the mean differences of Scr **(A)** overall effect, **(B)** subgroups according to stages, **(C)** subgroups according to baseline Scr levels, and **(D)** subgroups according to baseline blood glucose levels, with a random effects model.

In 12 of the studies (**Figure 8A**), which together represent 938 patients, Scr values were reported. Meta-analysis within the random-effects model showed that, compared with using CT, the combination of Shengjiang powder with CT resulted in lower Scr values [MD = -9.87, 95% CI (-13.48,-6.27), P <0.001]. Subgroup analysis results revealed that heterogeneity among subgroups did not decrease, and there was no statistical difference between the MDs of duration subgroups (**Supplementary Figure 1**, **Supplementary Table 4**). In the case of stage II/II patients and those with lower or higher baseline glucose levels, there was no statistically significant difference between the control group and the experimental groups (**Figures 8B, C**). In addition, we conducted an extra subgroup analysis based on the baseline Scr levels, and the results indicated that the efficacy of treatment in the group with more severe kidney impairment (Scr > 133μmol/L) was better than in the group with lighter impairment (Scr < 133μmol/L) (**Figure 8D**).

### 3.4 Meta-analysis of secondary outcomes

#### 3.4.1 FBG

**Figure 9:**
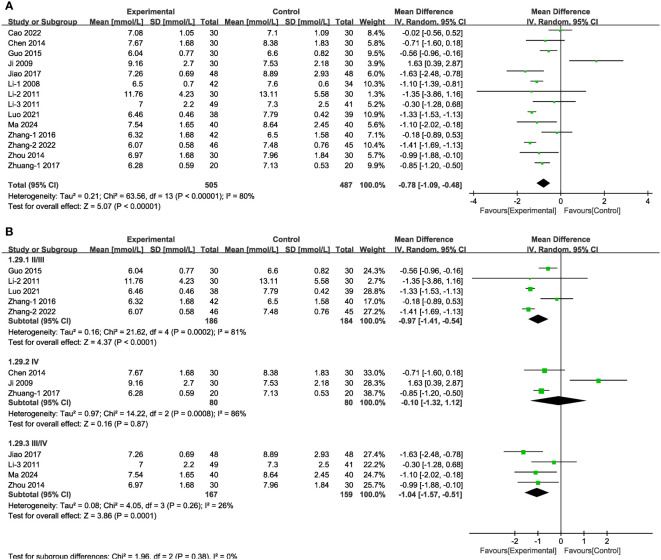
Forest plots illustrating the mean differences of FBG **(A)** overall effect, and **(B)** subgroups according to stages, with a random effects model.

In 14 of the studies (**Figure 9A**), which together represent 992 patients, FBG values were reported. Meta-analysis within the random-effects model indicated that the combination of TCM (including Shengjiang powder) and CT was more efficacious than was CT alone in decreasing FBG [MD = -0.78, 95% CI (-1.09,-0.48), P < 0.001]. Subgroup analysis results revealed that heterogeneity among subgroups was still significant. No significant difference existed among the varied duration and in the group stage IV (**Figure 9B**, **Supplementary Figure 1**, **Supplementary Table 4**).

#### 3.4.2 2hPG

**Figure 10:**
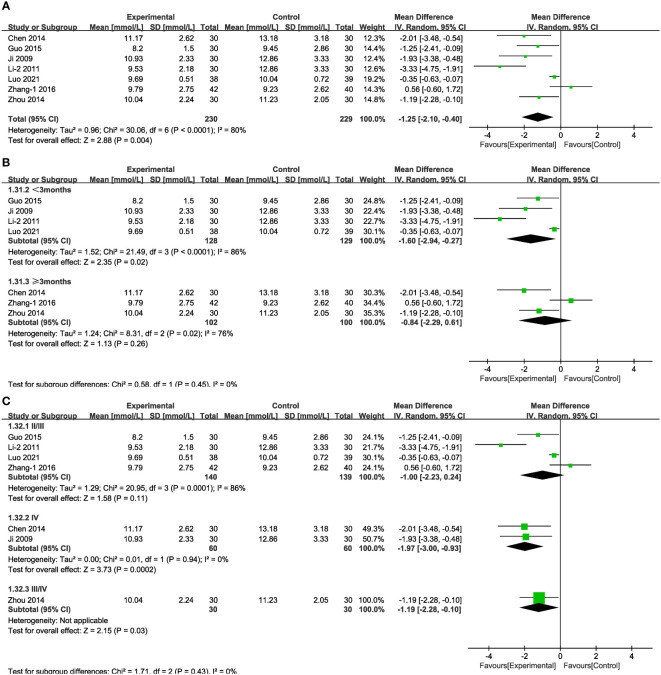
Forest plots illustrating the mean differences of 2hPG **(A)** overall effect, **(B)** subgroups according to durations, and **(C)** subgroups according to stages, with a random effects model.

In the case of seven of the studies (**Figure 10A**), representing 459 patients, the meta-analysis within the random-effects model indicated that the combination of TCM and CT was more efficacious than was CT alone in decreasing 2hPG [*MD* = -1.25, 95% CI (-2.10,-0.40), P < 0.01]. No source of heterogeneity was found through subgroup analysis. There was no statistical difference associated with the results between the experimental and control groups in stage IV and in the results obtained from treatment duration more than three months (**Figures 10B, C**).

#### 3.4.3 HbA1c

**Figure 11:**
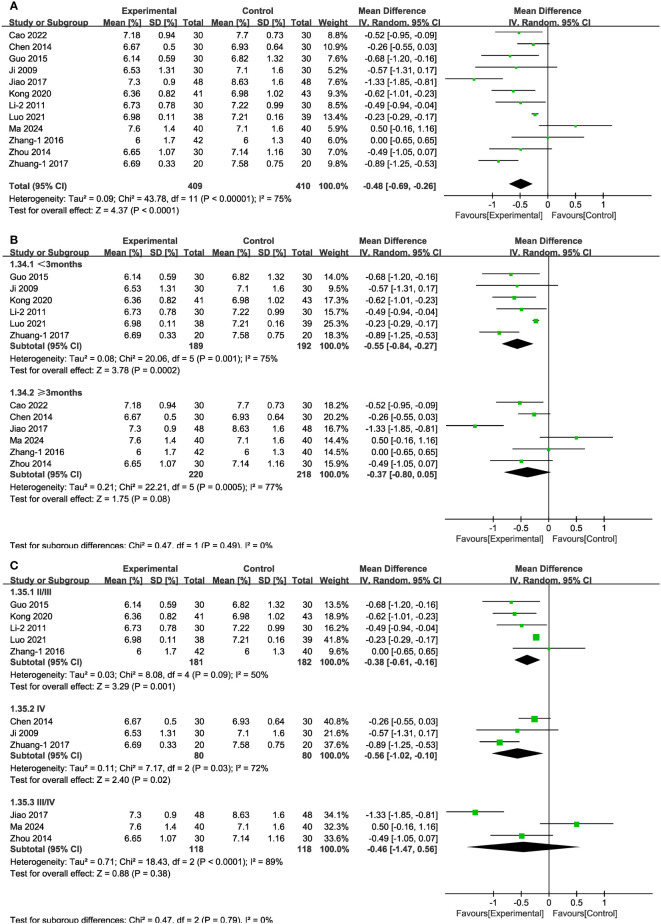
Forest plots illustrating the mean differences of HbA1c **(A)** overall effect, **(B)** subgroups according to durations, and **(C)** subgroups according to stages, with a random effects model.

In 12 of the studies (**Figure 11A**), which together represent 819 patients, HbA1c values were reported. Meta-analysis within the random-effects model showed that the combination of TCM and CT was more effective in lowering HbA1c than was CT alone [MD = -0.48, 95% CI (-0.69,-0.26), P < 0.001]. No source of heterogeneity was found through subgroup analysis. In stage III/IV subgroups and in the cases of treatment lasting more than three months, the difference between experimental and control groups was not statistically significant (**Figures 11B, C**).

#### 3.4.4 TC

**Figure 12:**
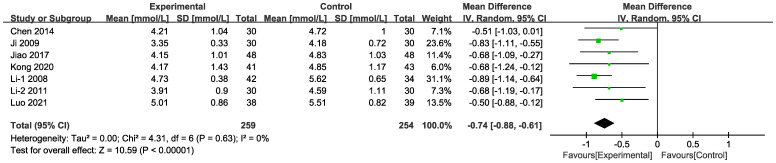
Forest plots illustrating the mean differences of TC.

In seven of the studies (**Figure 12**), included data on 513 patients, TC levels were measured. The results of the random-effects model suggest that the combined intervention was more effective than CT alone in terms of lowering TC [MD = -0.74, 95% CI (-0.88,-0.61), P < 0.001]. Differences in MD values among the subgroups were not statistically significant (**Supplementary Figure 2**, **Supplementary Table 4**).

#### 3.4.5 TG

**Figure 13:**
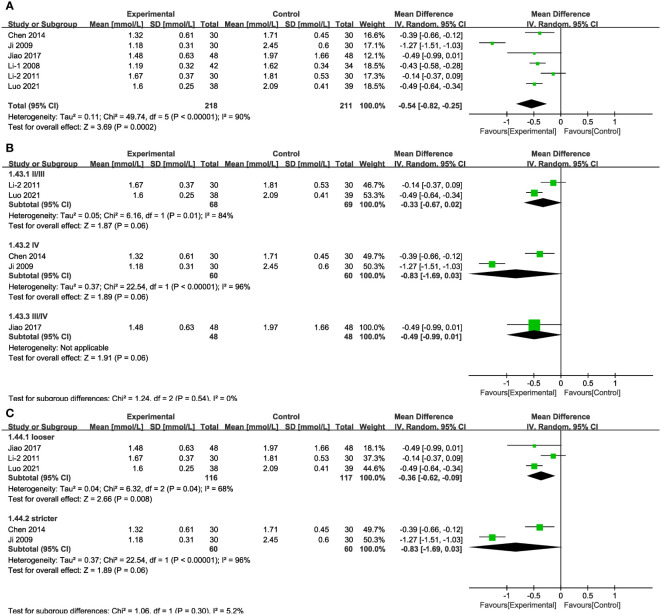
Forest plots illustrating the mean differences of TG **(A)** overall effect, **(B)** subgroups according to stages, and **(C)** subgroups according to blood glucose control levels, with a random effects model.

In six of the studies (**Figure 13A**), representing 429 patients, TG values were reported. The results of random-effects modelling suggest that the combined interventions were more effective than CT alone in lowering TG [MD = -0.54, 95% CI (-0.82,-0.25), P < 0.05]. No source of heterogeneity was found through subgroup analysis. Differences in MD values between subgroups based on treatment duration or baseline blood glucose levels were not statistically significant (**Supplementary Figure 2**, **Supplementary Table 4**), as was also the case in the varied stages and in the group with blood glucose levels strictly controlled (**Figures 13B, C**).

#### 3.4.6 Adverse events

**Figure 14:**
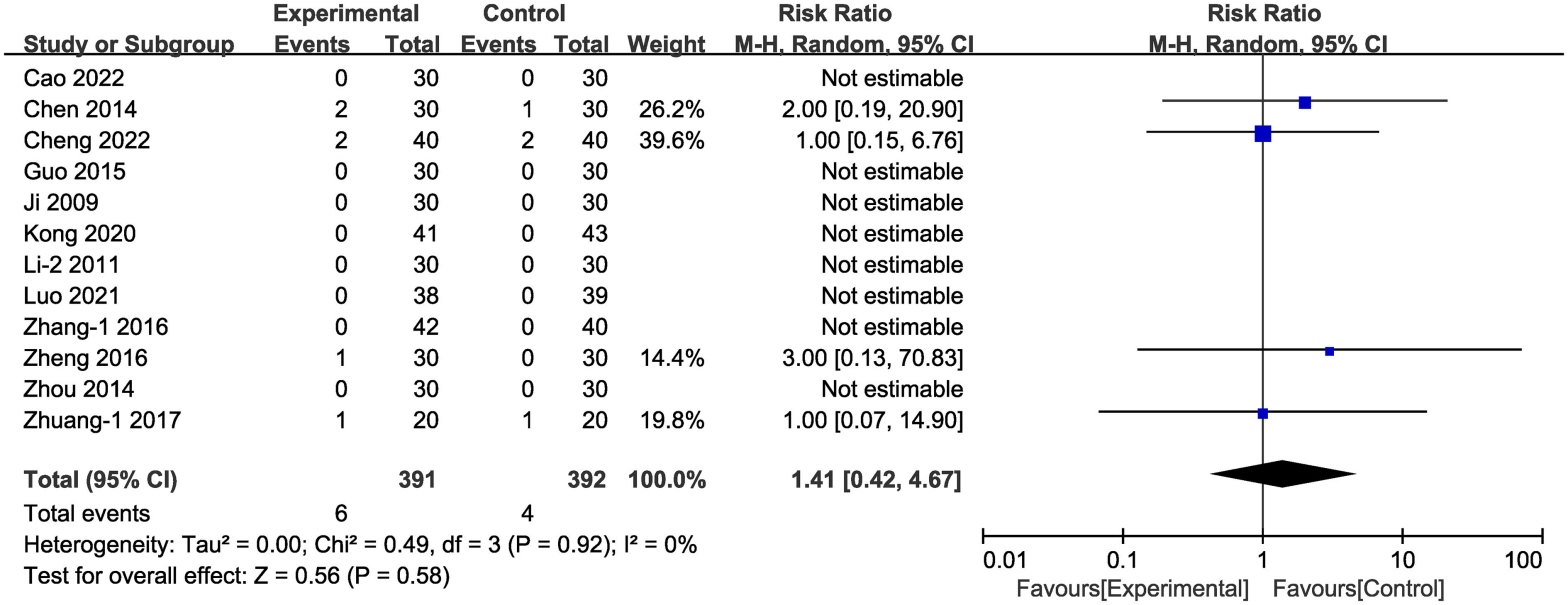
Forest plot of adverse events with a random effects model.

Adverse effects were documented in 13 of the studies (**Figure 14**). In four of these studies (38, 42, 49, 58) adverse events occurred, totally 10 individual cases. In the experimental group, the six cases of adverse effects were: three cases of nausea, two cases of mild diarrhea or abdominal discomfort, and two cases of mildly elevated aminotransferase. In the control group, the four cases of adverse effects were: one case of nausea, one case of dizziness, one case of dry coughing, one case of hypoglycemia. No adverse events were reported in the other nine studies. There was no statistically significant difference between adverse events rates in the experimental vs. the control groups [RR = 1.41, 95% CI (0.42,4.67), P = 0.58]. In one of the studies (56) the authors reported recurrence during follow-up, including 98 cases; and the recurrence rate in the experimental group (4.08%) was lower than that of the control group (18.37%).

### 3.5 Sensitivity analysis

Sensitivity analyses were performed by excluding each study from the included literature. With respect to the mALB results (P < 0.001; I2 = 82%), no heterogeneity was discovered (P = 0.41; I²=0%) after excluding one study (59) [MD = -16.63, 95% CI (-20.33,-12.94), P < 0.001]. In the case of TG (P < 0.001; I²=90%), heterogeneity was reduced (P = 0.17; I²=38%) once a particular study (50) was excluded [MD = -0.39, 95% CI (-0.51,-0.27), P < 0.001]. No obvious directional change occurred with respect to the other indicators, confirming that the results of our analysis were relatively robust.

### 3.6 Other analyses

#### 3.6.1 Publication bias assessment

Funnel plots were drawn for the following measures: clinical efficacy, 24-h urinary protein, BUN, Scr, FBG, and HbA1c. Partial asymmetry existed between the two sides of the funnel plots (**Supplementary Figure 3**), indicating that there may be publication bias and small sample effects (64). By applying Egger’s test, we detected possible publication bias with respect to clinical efficacy, 24-h urinary protein, BUN, and Scr (P < 0.05) (**Supplementary Figure 4**, **Supplementary Table 5**). The trim-and-fill method was applied to the effect size of these three indicators (**Supplementary Figure 5**, **Supplementary Table 6**), and the results confirmed that publication bias had no significant effect on these outcomes; thus the output of our analysis can be considered stable. Statistical significance with respect to clinical efficacy was apparent both before and after trimming and filling. As for 24-h urinary protein, BUN, and Scr, these parameters were not filled after linear iteration.

#### 3.6.2 GRADE

By evaluating the twelve outcomes with GRADEpro GPT, we found that UAER, mALB, BUN, Scr, 2hPG, HbA1c, TC, and TG were assessed as moderate due to the unclear risk of the studies. Clinical efficacy, 24-h urinary protein, BUN, Scr, and adverse events were ranked low due to inappropriate or unclear randomization, the presence of publication bias, or inaccuracy (**Supplementary Table 7**).

#### 3.6.3 *Post hoc* analysis

Subgroup analyses based on whether specific doses were mentioned, the differences in specific diagnostic criteria (**Table 1**), and the risk of bias of the studies, showed that none of them were the source of heterogeneity (**Supplementary Figures 6–8**, **Supplementary Table 4**). In the high-risk subgroups of 24-h urinary protein, mALB, FBG, and TG, and the other risk subgroup of 2hPG there was no statistical difference between the control group and the experimental group (**Supplementary Figure 8**, **Supplementary Table 4**).

## 4 Discussion

The results of our analysis of the relevant literature serve as evidence of the effectiveness of Shengjiang powder as a treatment of DKD, as indicated by the alleviation of key indicators of the disease: 24-h urinary protein and the degree of kidney damage. Moreover, the use of Shengjiang powder offers the additional benefit of supplementing the effects of oral hypoglycemic medications and insulin to control short-term blood glucose fluctuations and stabilize long-term blood glucose levels. Alongside CT, Shengjiang powder also has the potential to regulate blood lipid levels. The GRADE analysis demonstrated that the quality of the 12 disease indicators analyzed in this study varies from moderate to low.

Through subgroup analysis we uncovered additional important correlations. The beneficial effects of combined treatment—as reflected in lower levels of UAER, 2hPG, and HbA1c—were more pronounced in cases of shorter treatment duration. This finding is consistent with the principles of TCM, which advocate individualized medication adjustment and modification of the prescription based on improvements. Therefore, real-time evaluation is crucial during combined treatment to attain optimal clinical efficacy.

Shengjiang powder was found to be more effective in stage IV patients than in patients at earlier stages, based on the post-treatment levels of 24-h urinary protein, Scr, and 2hPG reported in the studies. However, the reverse was true for FBG values, which were lowered to greater effect in in stage II/III cases. To delve deeper into the relationship between the effectiveness of Shengjiang powder and disease severity, we conducted an additional subgroup analysis performed on baseline Scr levels. The finding showed that treatment that included Shengjiang powder apparently reduced Scr levels, particularly in patients with severe kidney impairment (higher baseline Scr), suggesting an underlying correlation between the benefits of Shengjiang powder for DKD patients and the degree of kidney dysfunction within a range. Based on the TCM view that of “long-standing diseases are bound to cause blood stasis” and the theory of “microscopic blockage and mass” (65), it is hypothesized that the effect of Shengjiang powder may involve the prominent presence of blood stasis factors and an expanded range of glomerulosclerosis in the latter stages of DKD.

Considering the potential relationship between blood glucose levels, the progression of DKD, and metabolic disturbances, subgroup analyses involving urinary protein, kidney function, and lipid indicators based on post-treatment HbA1c levels and baseline FBG levels were performed. The results suggested that stricter blood glucose control enhanced the effectiveness of Shengjiang powder in decreasing 24-h urinary protein excretion. In contrast, under more lax control of blood glucose control, the lipid-regulating effects of Shengjiang powder became apparent. In patients with higher baseline blood glucose levels (FBG > 11.1 mmol/L), TCM + CT interventions led to a more pronounced reduction in BUN and Scr levels. However, due to incomplete data on post-treatment HbA1c and baseline FBG levels in all studies, further analysis of other urinary protein and kidney function indicators was not feasible.

The level of urinary protein serves as a crucial marker for evaluating the severity of kidney injury in DKD, with reducing urinary albumin excretion rate being a pivotal aspect in preventing and delaying the progression of DKD (66). The United Kingdom Prospective Diabetes Study (UKPDS) highlighted that while patients with substantial urinary protein are relatively scarce, those with pronounced urinary protein face a mortality rate surpassing the progression rates of other kidney diseases (15). Given the current treatment limitations, inadequate control of urinary protein among DKD patients will likely lead to the majority of these cases progressing to ESRD within a short period. The patients treated in the studies we investigated predominantly fell within stage III (characterized by sustained mALB) and stage IV (marked by clinically evident urinary protein), encompassing the critical phases for reversing kidney damage. Meta-analysis results confirm that Shengjiang powder can reduce 24-h urinary protein, UAER, and mALB in stage III/IV DKD patients. In TCM, falling within the realm of “essence”, protein is regarded as the lucid yang that nourishes the organs. The body’s recovery of dispersing essence can be aided by raising the lucid yang with the help of stiff silkworm and cicada slough, aligning with fundamental research results (67–69).

Three large studies have illustrated that achieving blood glucose control targets can reverse substantial urinary protein in DKD patients (15, 70, 71), affirming that maintaining appropriate blood glucose levels and stringent blood glucose regulation can efficiently delay the progression of DKD. Our analysis of studies in which the focus has been on FBG, 2hPG, and HbA1c, in conjunction with the benefits of Shengjiang powder on BUN and Scr levels, shows that this herbal formula can play an adjuvant role for DKD to anti-hyperglycemia and stabilize blood glucose fluctuations.

Lipid abnormalities represent a significant factor in the progression of chronic kidney disease (72), with a particular emphasis on DM, which not only signifies a keynote metabolic disturbance but also serves as a critical risk factor for diabetic panvascular diseases. A key principle of TCM is that high lipid levels impair the flow of Qi movement, disrupting the normal diffusion of food and drink. Eventually, this dysfunction contributes to the deposition of fat (73), which congests vessel. This stagnation results in the accumulation of heat over time, which stresses or “burns” the blood vessel, leading to stasis throughout the body, as is manifest in DKD patients with hyperlipidemia and associated complications. Shengjiang powder is used to delay the development of the disease by purging the heat of collaterals (74); in other words, making possible the phenomenon of ascending clarity and descending turbidity, which, in conventional medical terms is described as the regulation of lipid metabolism. High TG levels in DM are a marker of insulin resistance (75). In our analysis of the relevant literature, the experimental groups tended to showed a more favorable outcome in terms of reducing TC and TG, suggesting a potential therapeutic effect for Shengjiang powder in ameliorating insulin resistance and averting the onset of cardiovascular diseases in patients with DKD.

We should point out that there were some limitations in our study. 1) The included studies were all Chinese studies; i.e., patients from other countries were not included. 2) Publication bias was suspected upon examination of the data, and the patient cohorts were generally small. Although trim-and-fill methods indicate minimal impacts of publication bias on the results, there may still be potential small-sample effects affecting the quality of the outcomes reported in these studies. 3) Randomization was inappropriate or not described in several studies, and blinding or allocation concealment was inadequately addressed in the majority of the papers, posing a certain risk of bias. 4) Most of the studies in our data set showed significant heterogeneity, which may stem from inconsistencies in baseline blood glucose levels, durations, stages, or interventions. Though we sought to probe the potential sources of heterogeneity, the inherent variances in study characteristics and definitions may hinder a comprehensive elucidation of all sources of heterogeneity. 5) Variability exists in the durations of the included studies, with not all studies reporting adverse event occurrences. Moreover, only two studies included a follow-up protocol; thus, the long-term prognosis of Shengjiang powder for DKD patients uncertain. The safety of this type of treatment requires further study before it can be confirmed. 7) Due to the intricate nature of TCM, it was impractical to launch an further analysis beyond the components of Shengjiang powder in the interventions.

## 5 Conclusion

Considering the results of our comprehensive analysis of the relevant literature, we can tentatively conclude that Shengjiang powder has the potential to supplement CT as an effective treatment delivering benefits for patients diagnosed with DKD. Specifically, Shengjiang powder, when combined with CT, appears to reduce the level of urinary protein, stabilize kidney function, and improve blood glucose and lipid metabolism. Considering the significant heterogeneity among studies and the limitation of the quality of the published studies, the conclusions we present here need to be further verified by more studies with larger samples (especially multi-center samples) and generally high-quality data.

## Data availability statement

The raw data supporting the conclusions of this article will be made available by the authors, without undue reservation.

## Author contributions

ZZ: Conceptualization, Data curation, Formal Analysis, Investigation, Methodology, Project administration, Visualization, Writing – original draft, Writing – review & editing. YL: Data curation, Investigation, Writing – original draft. JQ: Data curation, Writing – review & editing. WJ: Data curation, Writing – original draft. LC: Data curation, Writing – original draft. ZL: Supervision, Writing – review & editing. HC: Supervision, Writing – review & editing. SL: Methodology, Supervision, Writing – review & editing.

## References

[1] Anders H Huber TB Isermann B Schiffer M . CKD in diabetes: diabetic kidney disease versus nondiabetic kidney disease. Nat Rev Nephrol. (2018) 14:361–77. doi: 10.1038/s41581-018-0001-y 29654297

[2] Zhang J Su B Zhang J Guo X . Expert consensus on early prediction and diagnosis of diabetic kidney disease. Chin J Internal Med. (2021) 60:522–32. doi: 10.3760/cma.j.cn112138-20200603-00550 34058808

[3] Shaw JE Sicree RA Zimmet PZ . Global estimates of the prevalence of diabetes for 2010 and 2030. Diabetes Res Clin Pr. (2010) 87:4–14. doi: 10.1016/j.diabres.2009.10.007 19896746

[4] Whiting DR Guariguata L C. Weil and J . Shaw: IDF Diabetes Atlas: Global estimates of the prevalence of diabetes for 2011 and 2030. Diabetes Res Clin Pr. (2011) 94:311–21. doi: 10.1016/j.diabres.2011.10.029 22079683

[5] Ogurtsova K Da Rocha Fernandes JD Huang Y Linnenkamp U Guariguata L Cho NH . IDF Diabetes Atlas: Global estimates for the prevalence of diabetes for 2015 and 2040. Diabetes Res Clin Pr. (2017) 128:40–50. doi: 10.1016/j.diabres.2017.03.024 28437734

[6] Saeedi P Petersohn I Salpea P Malanda B Karuranga S Unwin N . Global and regional diabetes prevalence estimates for 2019 and projections for 2030 and 2045: Results from the International Diabetes Federation Diabetes Atlas, 9th edition. Diabetes Res Clin Pr. (2019) 157:107843. doi: 10.1016/j.diabres.2019.107843 31518657

[7] EGCDN . Expert consensus on multidisciplinary diagnosis, treatment, and management of diabetic nephropathy. Chin J Clin. (2020) 48:522–7. doi: 10.3969/j.issn.2095-8552.2020.05.006

[8] Ruiz-Ortega M Rodrigues-Diez RR Lavoz C Rayego-Mateos S . Special issue “Diabetic nephropathy: diagnosis, prevention and treatment. J Clin Med. (2020) 9:813. doi: 10.3390/jcm9030813 32192024 PMC7141346

[9] ADA . 11. Microvascular complications and foot care: standards of medical care in diabetes–2020. Diabetes Care. (2019) 43:S135–51. doi: 10.2337/dc20-S011 31862754

[10] Gross JL de Azevedo MJ Silveiro SP Canani LH Caramori ML Zelmanovitz T . Diabetic nephropathy: diagnosis, prevention, and treatment. Diabetes Care. (2005) 28:164–76. doi: 10.2337/diacare.28.1.164 15616252

[11] Scheen AJ Paquot N . [Management of hyperglycaemia of type 2 diabetes. Paradigm change according to the ADA-EASD consensus report 2018]. Rev Med Liege. (2018) 73:629–33. 30570234

[12] Alicic RZ Rooney MT Tuttle KR . Diabetic kidney disease. Clin J Am Soc Nephro. (2017) 12:2032–45. doi: 10.2215/CJN.11491116 PMC5718284 28522654

[13] Stenvinkel P Chertow GM Devarajan P Levin A Andreoli SP Bangalore S . Chronic inflammation in chronic kidney disease progression: role of Nrf2. Kidney Int Rep. (2021) 6:1775–87. doi: 10.1016/j.ekir.2021.04.023 PMC8258499 34307974

[14] Itoh Y Yasui T Kakizawa H Makino M Fujiwara K Kato T . The therapeutic effect of lipo PGE1 on diabetic neuropathy-changes in endothelin and various angiopathic factors. Prostaglandins Other Lipid Mediat. (2001) 66:221–34. doi: 10.1016/S0090-6980(01)00165-4 11577785

[15] Adler AI Stevens RJ Manley SE Bilous RW Cull CA Holman RR . Development and progression of nephropathy in type 2 diabetes: The United Kingdom Prospective Diabetes Study (UKPDS 64). Kidney Int. (2003) 63:225–32. doi: 10.1046/j.1523-1755.2003.00712.x 12472787

[16] CSN . Chinese guidelines for diagnosis and treatment of diabetic kidney disease. Chin J Nephrol. (2021) 37:255–304. doi: 10.3760/cma.j.cn441217-20201125-00041

[17] Thomas MC Brownlee M Susztak K Sharma K Jandeleit-Dahm KAM Zoungas S . Diabetic kidney disease. Nat Rev Dis Primers. (2015) 1:15018. doi: 10.1038/nrdp.2015.18 27188921 PMC7724636

[18] Kunz R Friedrich C Wolbers M Mann JFE . Meta-analysis: effect of monotherapy and combination therapy with inhibitors of the renin–angiotensin system on proteinuria in renal disease. Ann Intern Med. (2008) 148:30. doi: 10.7326/0003-4819-148-1-200801010-00190 17984482

[19] Maione A Navaneethan SD Graziano G Mitchell R Johnson D Mann JFE . Angiotensin-converting enzyme inhibitors, angiotensin receptor blockers and combined therapy in patients with micro- and macroalbuminuria and other cardiovascular risk factors: a systematic review of randomized controlled trials. Nephrol Dial Transpl. (2011) 26:2827–47. doi: 10.1093/ndt/gfq792 21372254

[20] Wu HY Huang JW Lin HJ Liao WC Peng YS Hung KY . Comparative effectiveness of renin-angiotensin system blockers and other antihypertensive drugs in patients with diabetes: systematic review and bayesian network meta-analysis. BMJ. (2013) 347:f6008–8. doi: 10.1136/bmj.f6008 PMC3807847 24157497

[21] Lin M Chiu Y Chang J Lin H Lee CT Chiu G . Association of prescribed Chinese herbal medicine use with risk of end-stage renal disease in patients with chronic kidney disease. Kidney Int. (2015) 88:1365–73. doi: 10.1038/ki.2015.226 26244923

[22] Yang X Hu C Wang S Chen Q . Clinical efficacy and safety of Chinese herbal medicine for the treatment of patients with early diabetic nephropathy. Medicine. (2020) 99:e20678. doi: 10.1097/MD.0000000000020678 32702818 PMC7373501

[23] Zhang L Miao R Yu T Wei R Tian F Huang Y . Comparative effectiveness of traditional Chinese medicine and angiotensin converting enzyme inhibitors, angiotensin receptor blockers, and sodium glucose cotransporter inhibitors in patients with diabetic kidney disease: A systematic review and network meta-analysis. Pharmacol Res. (2022) 177:106111. doi: 10.1016/j.phrs.2022.106111 35183713

[24] Huaming L Limei L . Analysis of TCM pathogenesis of diabetic nephropathy differing from other chronic kidney diseases. China J Chin Med. (2017) 32:2088–90. doi: 10.16368/j.issn.1674-8999.2017.11.546

[25] Zhang Y . A brief analysis of TCM etiology and pathogenesis of diabetic nephropathy. Guangming J Of Chin Med. (2010) 25:406–7. doi: 10.3969/j.issn.1003-8914.2010.03.043

[26] Tong X Zhou Q Zhao L Tian J . Experience on TCM syndrome differentiation and treatment of diabetic nephropathy. China J Tradit Chin Med. (2014) 29:144–6.

[27] Zhang L Cui D Yue D . Characteristics of Shengjiang powder for epidemic treatment. Chin J Basic Med Tradit Chin Med. (2022) 28:1845–7. doi: 10.19945/j.cnki.issn.1006-3250.20220309.005

[28] Lanzhi Z Limin D Wenting X Liying X Li W Yi C . Effect of Qingtou Jiedu decoction based on Shengjiang powder on clinical efficacy in treating sepsis of toxic heat syndrome. Chin J Integrated Tradit Western Med Intensive Crit Care. (2022) 29:22–6. doi: 10.3969/j.issn.1008-9691.2022.01.005

[29] Xiong J Zhao X Zhang Q . Yinqiao Mabo powder and Shengjiang powder combined with acupoint application were used to treat 50 children with upper airway cough syndrome. Jiangxi J Tradit Chin Med. (2022) 53:53–5.

[30] CDS . Expert consensus on prevention and treatment of diabetic nephropathy (2014 edition). Chin J Diabetes. (2014) 6:792–801. doi: 10.3760/cma.j.issn.1674-5809.2014.11.004

[31] Mogensen CE . Microalbuminuria, blood pressure and diabetic renal disease: origin and development of ideas. Diabetologia. (1999) 42:263–85. doi: 10.1007/s001250051151 10096778

[32] Zhen X . Guiding principles for clinical research of New Traditional Chinese Medicine (Trial). Beijing: China Medical Science and Technology Press (2002).

[33] ADA . Standards of medical care in diabetes—2010. Diabetes Care. (2010) 33:S11–61. doi: 10.2337/dc10-S011 PMC2797382 20042772

[34] S. EGKCQCC . Guidelines for early screening, diagnosis, prevention and treatment of chronic kidney disease (2022 Edition). Chin J Nephrol. (2022) 38:453–64. doi: 10.3760/cma.j.cn441217-20210819-00067

[35] Sterne J Savović J Page MJ Elbers RG Blencowe NS Boutron I . RoB 2: a revised tool for assessing risk of bias in randomised trials. BMJ. (2019) 366:l4898. doi: 10.1136/bmj.l4898 31462531

[36] Zhang T . Rational use of statistical models for traditional meta-analysis. Chin J Hosp Stat. (2023) 30:299–303,308. doi: 10.3969/j.issn.1006-5253.2023.04.012

[37] Zhang T Zhang S . How to compare summary estimates of different subgroups in meta-analysis. Chin J Evidence-Based Med. (2017) 17:1465–70.

[38] Cheng L Zhang Z Ma J Wang Q Liu H Luo W . Clinical efficacy of modified Shengjiang powder in the adjuvant treatment of patients with stage IV diabetic nephropathy. J Tianjin Univ Tradit Chin Med. (2022) 41:182–7. doi: 10.11656/j.issn.1673-9043.2022.02.13

[39] Zhuang J Luo C Bai Y Yang J Li B . Clinical effect of Wenshen granule in the treatment of diabetic nephropathy stage IV of Yang deficiency and blood stasis type. Chin J Modern Drug Appl. (2020) 14:200–2. doi: 10.14164/j.cnki.cn11-5581/r.2020.07.091

[40] Bian H . Clinical Study of TangShenShengQingJiangZhuo granules impact on CysC and UMA in diabetic nephropathy proteinuria period. HUCM, Henan, China: Henan University of Traditional Chinese Medicine (2015).

[41] Zhang S Yang C . Effect of Xinwei Tongxuan on high-sensitivity C-reavtive protein and 24h of Urine microalbumin with diabetic nephropathy. Clin J Tradit Chin Med. (2016) 28:366–8. doi: 10.16448/j.cjtcm.2016.0135

[42] Zhuang K Yang H Li L Li Y Han J Yang H . Clinical study on treatment of stage IV diabetic nephropathy with combine traditional Chinese and western medicine. Acta Chin Med. (2017) 32:1172–4. doi: 10.16368/j.issn.1674-8999.2017.07.308

[43] Zhou Y . DN shengqingjiangzhuo particles Clinical Intervention Research of diabetic nephropathy proteinuria PRA and Ang-II. HUCM, Henan, China: Henan University of Traditional Chinese Medicine (2014).

[44] Ma X Guo N Wang Y Yin X Cui J Yin Y . Buyang Huanwu decoction combined with Shengjiang powder in the treatment of diabetic kidney disease. Chin Med Modern Distance Educ China. (2024) 22:60–2.

[45] Cao X . "Turbidity-toxin"Huazhuo Jiedu Prescription Based onKidney Disease(Stagnation Heat Syndrome)withClinical Study on Intervention of Type 2 Diabetic. TUTCM, Tianjin, China: Tianjin University of Traditional Chinese Medicine (2022). doi: 10.27368/d.cnki.gtzyy.2022.000139

[46] Kong Y . Clinical Study of JiaWeiShengJiang Powder in the treatment of Early Diabetic Kidnev Disease and its effect on Oxidative Stress. HUCM, Hebei, China: Hebei University of Traditional Chinese Medicine (2020).

[47] Jiao D Liu A Wang X Du F Li M Li M . Clinical observation of Tangshen Shengqing Jiangzhuo Particles in treating diabetic nephropathy with proteinuria. Shanghai J Tradit Chin Med. (2017) 51:50–3. doi: 10.16305/j.1007-1334.2017.05.015

[48] Li X . Clinical Study of Sheng Qing Jiang Zhu prescription on third stage of diabetic nephropathy. HUCM, Henan, China: Henan University of Traditional Chinese Medicine (2011).

[49] Chen W Tang W Lu X Liang Y Hu J . Effect of Yiqi Wenyang Xiazhuo decoction in the treatment of clinical diabetic nephropathy. New Chin Med. (2014) 46:149–51. doi: 10.13457/j.cnki.jncm.2014.02.042

[50] Ji Z . Clinical Study of Liu Huang Tang Shen Kang prescription on fourth stage of diabetic nephropathy. HUCM, Henan, China: Henan University of Traditional Chinese Medicine (2009).

[51] Luo X . Clinical study of saving essence and dispelling turbidity therapy on the treatment of diabetic nephropathy III (spleen and kidney qi deficiency syndrome). CDUTCM, Chengdu, China: Chengdu University of Traditional Chinese Medicine (2021).

[52] Li J . Observation of the clinical effect of Shengjiang Guiling decoction on the treatment of diabetic nephropathy. Hebei J Tradit Chin Med. (2008) 06:573–4.

[53] Guo J . Shengqingjiangzhuo method for early diabetic nephropathy junction of phlegm and blood stasis, clinical efficacy research Guo Jiangcui(Internal Medicine of Traditional Chinese Medicine). HUCM, Henan, China: Henan University of Traditional Chinese Medicine (2015).

[54] Zhang Y Ren H Zhou Q Wang J . Clinical observation of modified Shengjiang powder in the treatment of diabetic nephropathy. J Pract Tradit Chin Med. (2022) 38:1725–7.

[55] Zhao L Ma J Liu H Feng R Luo W Zhang Z . Clinical study on the treatment of diabetic nephropathy with modified shengjiang powder. Internal Med China. (2022) 17:145–148+190. doi: 10.16121/j.cnki.cn45-1347/r.2022.02.05

[56] Gao J Yang H Wang F Pang L Su Z Yang H . Effect of Guiqi Shengjiang powder on early diabetic nephropathy of type 2 diabetes mellitus. J Hebei Tradit Chin Med. (2013) 28:22–3. doi: 10.16370/j.cnki.13-1214/r.2013.03.010

[57] Liu C . Modified Shengjiang powder combined with western medicine in treating diabetic nephropathy control observation. J Pract Tradit Chin Internal. (2012) 26:46–7.

[58] Zheng Y . Shengqingjiangzhuo formula for diabetic nephropathy renin angiotensin clinical intervention studies. HUCM, Henan, China: Henan University of Traditional Chinese Medicine (2016).

[59] Tang X . 76 cases of diabetic nephropathy were treated with modified Shengjiang powder. Chin J Exp Tradit Med Formulae. (2011) 17:247–8. doi: 10.13422/j.cnki.syfjx.2011.10.007

[60] Li X Ma X Zhang S Du C Li C . Effect of Buyang Huanwu decoction and Shengjiang powder on diabetic nephropathy. J Sichuan Tradit Chin. (2011) 29:58–9.

[61] Kidney Disease: Improving Global Outcomes (KDIGO) Diabetes Work Group . KDIGO 2020 clinical practice guideline for diabetes management in chronic kidney disease. Kidney Int. (2020) 98:S1–115. doi: 10.1016/j.kint.2020.06.019 32998798

[62] KDOQI . KDOQI clinical practice guidelines and clinical practice recommendations for diabetes and chronic kidney disease. Am J Kidney Dis. (2007) 49:S12–154. doi: 10.1053/j.ajkd.2006.12.005 17276798

[63] American Diabetes Association . (9) Microvascular complications and foot care. Diabetes Care. (2015) 38 Suppl:S58–66. doi: 10.2337/dc15-S012 25537710

[64] Zhang T . Appropriate use of funnel plots for traditional meta-analysis. Chin J Hosp Stat. (2023) 30:304–8. doi: 10.3969/j.issn.1006-5253.2023.04.013

[65] Wen L . A brief analysis of professor Lu Renhe's medical case of "six pairs of treatment" for diabetic nephropathy. BUCM, Beijing, China: Beijing University of Traditional Chinese Medicine (2016).

[66] Oshima M Shimizu M Yamanouchi M Toyama T Hara A Furuichi K . Trajectories of kidney function in diabetes: a clinicopathological update. Nat Rev Nephrol. (2021) 17:740–50. doi: 10.1038/s41581-021-00462-y 34363037

[67] Yu J Wang Q Yu H . Effects of Shengjiangsan on expression of NF-κB in rats with mesangial proliferative glomerulonephritis. Chin J Of Exp Tradit Med Formulae. (2011) 17:190–3. doi: 10.3969/j.issn.1005-9903.2011.10.057

[68] Bao H Chen R Pan X Huang W Sun H Wu Y . Effect of curcumin on human mesangial cell growth cultured *in vitro* . Acta Universitatis Medicinae Nanjing. (2003) 23:238–239,274. doi: 10.3969/j.issn.1007-4368.2003.03.016

[69] Bao H Chen R Guo M Huang S Zhang A Fei L . The effect of curcumin on the ultrastructure of kidney and cell proliferation in nephrotoxic sera nephritis rats. Jiangsu Med. (2004) 30:104–6. doi: 10.3969/j.issn.0253-3685.2004.02.009 15339499

[70] Agrawal L Azad N Bahn GD Reaven PD Hayward RA Reda DJ . Intensive glycemic control improves long-term renal outcomes in type 2 diabetes in the veterans affairs diabetes trial (VADT). Diabetes Care. (2019) 42:e181–2. doi: 10.2337/dc19-0891 PMC6804611 31548245

[71] Wong MG Perkovic V Chalmers J Woodward M Li Q Cooper ME . Long-term benefits of intensive glucose control for preventing end-stage kidney disease: ADVANCE-ON. Diabetes Care. (2016) 39:694–700. doi: 10.2337/dc15-2322 27006512

[72] CDS . Guideline for the prevention and treatment of type 2 diabetes mellitus in China (2020 edition). Chin J Diabetes. (2021) 13:315–409. doi: 10.3760/cma.j.cn115791-20210221-00095

[73] Zhen W Su H . 60 cases of hyperlipemia were treated with Shengjiang powder. Shaanxi J Of Tradit Chin Med. (2010) 31:1486–7. doi: 10.3969/j.issn.1000-7369.2010.11.033

[74] Tang S Liu C . The mechanism of "Blood stasis and heat" in Traditional Chinese Medicine: Treatment of internal injuries and Miscellaneous diseases – the third part of the academic experience of master Zhou Zhongying. Jiangsu J Tradit Chin Med. (2014) 8:1–4.

[75] Yang J . Effect of modified Shengjiang powder on adiponectin in patients with type 2 diabetes mellitus complicated with dyslipidemia. Chin J Basic Med Tradit Chin Med. (2012) 18:88–90. doi: 10.16254/j.cnki.53-1120/r.2012.09.008

