# Supplementary material for: Efficacy and safety of the traditional Chinese formula Shengjiang powder combined with conventional therapy in the treatment of diabetic kidney disease: a systematic review and meta-analysis
Source: Front Endocrinol (Lausanne). 2024 Jul 23;15:1400939. doi: 10.3389/fendo.2024.1400939 (PMC11300290; doi:10.3389/fendo.2024.1400939)
Supplement: Supplementary file 1 [file DataSheet_1.docx]

**Supplemental Table 1.** Search strategy.

| Search terms | | | |
| --- | --- | --- | --- |
| Diabetic Kidney Disease | Chinese | MeSH | 糖尿病肾病 |
|  |  | Free | 糖尿病性肾病, 早期糖尿病肾病, 糖尿病肾损伤, 糖尿病肾脏病, 糖尿病肾脏疾病, 糖尿病性肾小球硬化症, 糖尿病肾小球疾病, 结节性肾小球硬化症, 消渴, 消渴肾病, 消渴病肾病, 溺毒, 水肿, 尿浊, 关格 |
|  | English | MeSH | Diabetic Nephropathies |
|  |  | Free | diabetic nephropathy, Diabetic nephropathies, Diabetes nephropathy, Diabetic nephrosis, Diabetes nephrosis, Diabetic nephrology, Nephropathies, Diabetic, Nephropathy, Diabetic, Diabetic Nephropathy, DN, Diabetic Kidney Disease, Diabetic Kidney Diseases, Kidney Disease, Diabetic, Kidney Diseases, Diabetic, DKD, diabetic renal disease, Diabetic Glomerulosclerosis, Glomerulosclerosis, Diabetic, Intracapillary Glomerulosclerosis, intercapillary glomerulosclerosis, Nodular Glomerulosclerosis, Glomerulosclerosis, Nodular, Kimmelstiel-Wilson Syndrome, Kimmelstiel Wilson Syndrome, Syndrome, Kimmelstiel-Wilson, Kimmelstiel-Wilson Disease, Kimmelstiel Wilson Disease |
|  | Expanded P | Diabetes mellitus | diabetes mellitus, Diabetes Complications |
|  |  | Kidney injury | Renal function, kidney injury, renal damage, ESRD, CKD, chronic kidney disease, renal fibrosis, renal interstitial fibrosis, glomerular filtration rate |
| Shengjiang powder | Chinese | MeSH | 升降散 |
|  |  | Free | 升降汤 |
|  | Expanded I | Component | 僵蚕, 蝉蜕, 姜黄, 大黄 |
|  |  | Treatment principles and methods | 郁火, 郁热, 火郁发之, 升清降浊, 清宣郁热, 宣通气机, 透邪解郁, 开郁降气, 清热透邪 |
|  | English | MeSH | Shengjiang Powder |
|  |  | Free | Shengjiangsan, Shengjiang powders, SJP |
|  | Expanded I | Traditional Chinese medicine | Chinese herbal medicine, Traditional Chinese Medicine |
|  |  | Component | Stiff Silkworm, Bombyx Batryticatus, muscardine silkworm, mulberry silkworm, batryticated silkworm, Batryticatus Bombyx, bombyx cum batryte, silkworm, silkworm, Bombyx |
|  |  |  | cicada slough, cicada slough, Periostracum Cicadae, Cicadae Periostracum, cicada shell, periostracum cicada, cicada ecdysis, periostracum cicadae |
|  |  |  | turmeric, curcuma, turmerol, Curcuma longa, Curcuma longa Linn, curcumin, rhizoma curcumae longae, turmerone |
|  |  |  | Rhubarb, pieplant, rheum, rheum officinale, Radix Et Rhizoma Rhei, rheum officinale baill, rheum officinale baillon, Rhizoma Rhei, rhizoma phei, rhubarb horse-tails, Rhei Radix et Rhizoma, medicinal rhubarb, Da Huang, emodin, rheochrysin |
|  |  | Treatment principles and methods | depressed fire, fire depression, heat depression, fire depression is treated by effusion, upbear the clear and downbear the turbid, clearing and diffusing stagnant heat, unblocking qi movement, removing pathogenic factors and releasing stagnation, breaking stagnation and directing qi downwards, clearing heat and removing pathogenic factors |
| Example of search formula (Cochrane) | ID Search Hits #1 MeSH descriptor: [Diabetic Nephropathies] explode all trees 1807 #2 (diabetic nephropathy):ti,ab,kw OR (Diabetic nephropathies):ti,ab,kw OR (Diabetes nephropathy):ti,ab,kw OR (Diabetic nephrosis):ti,ab,kw OR (Diabetes nephrosis):ti,ab,kw 5906 #3 (Diabetic nephrology):ti,ab,kw OR (Nephropathies, Diabetic):ti,ab,kw OR (Nephropathy, Diabetic):ti,ab,kw OR (Diabetic Nephropathy):ti,ab,kw OR (DN):ti,ab,kw 6204 #4 (Diabetic Kidney Disease):ti,ab,kw OR (Diabetic Kidney Diseases):ti,ab,kw OR (Kidney Disease, Diabetic):ti,ab,kw OR (Kidney Diseases, Diabetic):ti,ab,kw OR (DKD):ti,ab,kw 3395 #5 (diabetic renal disease):ti,ab,kw OR (Diabetic Glomerulosclerosis):ti,ab,kw OR (Glomerulosclerosis, Diabetic):ti,ab,kw OR (Intracapillary Glomerulosclerosis):ti,ab,kw OR (intercapillary glomerulosclerosis):ti,ab,kw 2591 #6 (Nodular Glomerulosclerosis):ti,ab,kw OR (Glomerulosclerosis, Nodular):ti,ab,kw OR ("Kimmelstiel-Wilson syndrome"):ti,ab,kw OR (Kimmelstiel Wilson Syndrome):ti,ab,kw OR (Syndrome, Kimmelstiel-Wilson):ti,ab,kw 1 #7 (Kimmelstiel-Wilson Disease):ti,ab,kw OR (Kimmelstiel Wilson Disease):ti,ab,kw OR (diabetes mellitus):ti,ab,kw OR (Diabetes Complications):ti,ab,kw OR (Renal function):ti,ab,kw 105046 #8 (Kimmelstiel-Wilson Disease):ti,ab,kw OR (Kimmelstiel Wilson Disease):ti,ab,kw 1 #9 (kidney injury):ti,ab,kw OR (renal damage):ti,ab,kw OR (ESRD):ti,ab,kw OR (CKD):ti,ab,kw OR (chronic kidney disease):ti,ab,kw 26230 #10 (renal fibrosis):ti,ab,kw OR (renal interstitial fibrosis):ti,ab,kw OR (glomerular filtration rate):ti,ab,kw 13250 #11 #1 or #2 or #3 or #4 or #5 or #6 or #7 or #9 or #10 127759 #12 (Shengjiang Powder) OR (Shengjiangsan) OR (Shengjiang powders) OR (SJP) 71 #13 (Bombyx Batryticatus):ti,ab,kw OR (muscardine silkworm):ti,ab,kw OR (mulberry silkworm):ti,ab,kw OR (batryticated silkworm):ti,ab,kw OR (Batryticatus Bombyx):ti,ab,kw 8 #14 (bombyx cum batryte):ti,ab,kw OR (silkworm):ti,ab,kw OR (silkworm, Bombyx):ti,ab,kw OR (cicada slough):ti,ab,kw OR (cicada slough):ti,ab,kw 31 #15 (Periostracum Cicadae):ti,ab,kw OR (Cicadae Periostracum):ti,ab,kw OR (cicada shell):ti,ab,kw OR (periostracum cicada):ti,ab,kw OR (cicada ecdysis):ti,ab,kw 2 #16 (periostracum cicadae):ti,ab,kw OR (turmeric):ti,ab,kw OR (curcuma):ti,ab,kw OR (turmerol):ti,ab,kw OR (Curcuma longa):ti,ab,kw 1045 #17 (Curcuma longa Linn):ti,ab,kw OR (curcumin):ti,ab,kw OR (rhizoma curcumae longae):ti,ab,kw OR (turmerone):ti,ab,kw OR ("rhubarb"):ti,ab,kw 2005 #18 (pieplant):ti,ab,kw OR ("Rheum"):ti,ab,kw OR (rheum officinale):ti,ab,kw OR (Radix Et Rhizoma Rhei):ti,ab,kw OR (rheum officinale baill):ti,ab,kw 283 #19 (rheum officinale baillon):ti,ab,kw OR (Rhizoma Rhei):ti,ab,kw OR (rhizoma phei):ti,ab,kw OR (rhubarb horse-tails):ti,ab,kw OR (Rhei Radix et Rhizoma):ti,ab,kw 22 #20 (medicinal rhubarb):ti,ab,kw OR (Da Huang):ti,ab,kw OR (emodin):ti,ab,kw OR (rheochrysin):ti,ab,kw OR (Stiff Silkworm):ti,ab,kw 79 #21 #12 or #13 or #14 or #15 or #16 or #17 or #18 or #19 or #20 2980 #22 (depressed fire) OR (fire depression) OR (heat depression) OR (fire depression is treated by effusion) OR (upbear the clear and downbear the turbid) 543 #23 (clearing and diffusing stagnant heat) OR (unblocking qi movement) OR (removing pathogenic factors and releasing stagnation) OR (breaking stagnation and directing qi downwards) OR (clearing heat and removing pathogenic factors) 4 #24 #21 or #22 or #23 3525 #25 #11 and #24 357 | | |
|  |  |  |  |
|  |  |  |  |
|  |  |  |  |
|  |  |  |  |
|  |  |  |  |
|  |  |  |  |
|  |  |  |  |
|  |  |  |  |
|  |  |  |  |
|  |  |  |  |
|  |  |  |  |
|  |  |  |  |
|  |  |  |  |
|  |  |  |  |
|  |  |  |  |
|  |  |  |  |
|  |  |  |  |
|  |  |  |  |
|  |  |  |  |
|  |  |  |  |
|  |  |  |  |
|  |  |  |  |
|  |  |  |  |
|  |  |  |  |
|  |  |  |  |
|  |  |  |  |
|  |  |  |  |
|  |  |  |  |
|  |  |  |  |
|  |  |  |  |
|  |  |  |  |
|  |  |  |  |
|  |  |  |  |
|  |  |  |  |
|  |  |  |  |
|  |  |  |  |
|  |  |  |  |
|  |  |  |  |
|  |  |  |  |
|  |  |  |  |
|  |  |  |  |
|  |  |  |  |
|  |  |  |  |
|  |  |  |  |
|  |  |  |  |
|  |  |  |  |
|  |  |  |  |
|  |  |  |  |
|  |  |  |  |
|  |  |  |  |
|  |  |  |  |

**Supplemental Table 2.** Descriptive statistics.

|  | (1) | (2) | (3) | (4) | (5) |
| --- | --- | --- | --- | --- | --- |
| VARIABLES | N | mean | sd | min | max |
| Stiff silkworm | 16 | 11.19 | 3.250 | 6 | 20 |
| Cicada slough | 16 | 10.94 | 3.750 | 6 | 15 |
| Rheum officinale(root and rhizome) | 16 | 7.938 | 3.235 | 5 | 15 |
| Curcuma longa (rhizome) | 16 | 9.938 | 2.768 | 6 | 15 |

**Supplemental Table 3.** The specific composition and daily dose of TCM in each intervention.

| Studies | Intervention | Composition |  |
| --- | --- | --- | --- |
| Ma 2024 | Buyang-Huanwu Decoction + Shengjiang powder | Stiff silkworm 12g, Cicada slough 12g, Rheum officinale(root and rhizome) 6g, Curcuma longa(rhizome) 12g, Astragalus mongholicus(root) 40g, Angelica sinensis(root) 15g, Rhizoma Ligustici(chuan xiong) 15g, Lumbricus 15g, Prunus persica(seed) 10g, Prunus persica(root) 15g, Carthamus tinctorius(flower) 10g | |
| Zhao 2022 | Modified Shengjiang powder | Stiff silkworm 9g, Cicada slough 9g, Rheum officinale(root and rhizome) 6g, Curcuma longa(rhizome) 12g, Prunus persica(seed) 9g, Leech 6g, Tabanus 6g, Centella asiatica 15g, Potentilla discolor 30g | |
| Cheng 2022 | Modified Shengjiang powder | Stiff silkworm 9g, Cicada slough 9g, Rheum officinale(root and rhizome) 6g, Curcuma longa(rhizome) 12g, Astragalus mongholicus(root) 30g, Prunus persica(seed) 9g, Leech 6g, Tabanus 6g, Centella asiatica 15g, Potentilla discolor 30g | |
| Zhang-2 2022 | Variant Shengjiang powder | Stiff silkworm 9g, Cicada slough 9g, Rheum officinale(root and rhizome) 10g, Curcuma longa(rhizome) 10g, Astragalus mongholicus(root) 15g, Atractylodes macrocephala(rhizome) 20g, Euryale ferox(seed) 12g, Rubus chingii(unripe fruit) 10g | |
| Cao 2022 | Huazhuo-Jiedu prescription | Stiff silkworm 10g, Cicada slough 6g, Rheum officinale(root and rhizome) 6g, Curcuma longa(rhizome) 15g, Bupleurum chinense(root) 12g, Scutellaria baicalensis(root) 15g, Coptis chinensis(rhizome) 10g, Pinellia ternata(processed tuber) 15g, Zingiber officinale(dried rhizome) 6g, Citrus trifoliata(unripe fruit) 10g, Paeonia lactiflora(root) 15g, Eupatorium fortunei 20g | |
| Luo 2021 | Modified Shengjiang powder | Stiff silkworm 20g, Cicada slough 15g, Rheum officinale(root and rhizome) 5g, Curcuma longa(rhizome) 10g, Astragalus mongholicus(root) 20g, Poria 15g, Atractylodes macrocephala(rhizome) 10g, Codonopsis pilosula(root) 30g, Glycyrrhiza uralensis(root and rhizome) 6g, Dioscorea oppositifolia(tuber) 20g | |
| Kong 2020 | Modified Shengjiang powder | Stiff silkworm 15g, Cicada slough 15g, Rheum officinale(root and rhizome) 5g, Curcuma longa(rhizome) 12g, Astragalus mongholicus(root) 30g, Rehmannia glutinosa(tuber) 15g, Coptis chinensis(rhizome) 12g, Smilax glabra(rhizome) 30g | |
| Zhuang-2 2020 | Wenshen Granules | Stiff silkworm 10g, Cicada slough 10g, Rheum officinale(root and rhizome) 15g, Curcuma longa(rhizome) 10g, Astragalus mongholicus(root) 30g, Atractylodes macrocephala(rhizome) 20g, Rosa laevigata(ripe fruit) 10g, Cornus officinalis(ripe fruit) 10g, Salvia miltiorrhiza(root and rhizome) 15g | |
| Zhuang-1 2017 | Guiqi-Shengjiang Granules | Stiff silkworm 10g, Cicada slough 15g, Rheum officinale(root and rhizome) 9g, Curcuma longa(rhizome) 9g, Astragalus mongholicus(root) 60g, Poria 30g, Angelica sinensis(root) 15g, Turtle shell 15g, Lumbricus 9g, Euonymus alatus(branches) 20g | |
| Jiao 2017 | Tangnshen-Shengqing-Jiangzhuo Particles | Stiff silkworm, Cicada slough, Rheum officinale(root and rhizome), Curcuma longa(rhizome), Astragalus mongholicus(root), Poria, Angelica sinensis(root), Dioscorea oppositifolia(tuber), Rehmannia glutinosa(cooked tuber), Achyranthes bidentata(root), Paeonia suffruticosa(root bark), Alisma plantago-aquatica(tuber), Abelmoschus moschatus, Coptis chinensis(rhizome), Centella asiatica, Euonymus alatus(branches) | |
| Zhang-1 2016 | Tangshenning Caps | Stiff silkworm, Cicada slough, Rheum officinale(root and rhizome), Curcuma longa(rhizome), Astragalus mongholicus(root), Eucommia ulmoides(bark), Cornus officinalis(ripe fruit), Salvia miltiorrhiza(root and rhizome), Arctium lappa(ripe fruit), Pueraria(root), Oldenlandia diffusa | |
| Zheng 2016 | Shengqing-Jiangzhuo Decoction | Stiff silkworm 10g, Cicada slough 6g, Rheum officinale(root and rhizome) 5g, Curcuma longa(rhizome) 6g, Astragalus mongholicus(root) 30g , Pseudostellaria heterophylla(root) 30g, Atractylodes macrocephala(rhizome) 20g, Glycyrrhiza uralensis(root and rhizome) 6g, Angelica sinensis(root) 15g, Rhizoma Ligustici(chuan xiong) 15g, Panax notoginseng(powdered) 6g, Abelmoschus moschatus 15g, Euonymus alatus(branches) 30g, Coptis chinensis(rhizome) 10g | |
| Bian 2015 | Tangshen-Shengqing-Jiangzhuo Granules | Stiff silkworm, Cicada slough, Rheum officinale(root and rhizome), Curcuma longa(rhizome), Astragalus mongholicus(root), Poria, Alisma plantago-aquatica(tuber), Angelica sinensis(root), Rehmannia glutinosa(cooked tuber), Dioscorea oppositifolia(tuber), Moutan, Achyranthes bidentata(root), Coptis chinensis(rhizome), Abelmoschus moschatus, Centella asiatica, Euonymus alatus(branches) | |
| Guo 2015 | Shengqing-Jiangzhuo Decoction | Stiff silkworm, Cicada slough, Rheum officinale(root and rhizome), Curcuma longa(rhizome), Astragalus mongholicus(root), Atractylodes macrocephala(rhizome), Glycyrrhiza uralensis(root and rhizome), Dioscorea oppositifolia(tuber), Rhizoma Ligustici(chuan xiong), Salvia miltiorrhiza(root and rhizome), Abelmoschus moschatus, Euonymus alatus(branches), Coptis chinensis(rhizome), Centella asiatica | |
| Chen 2014 | Yiqi-Wenyang-Xiezhuo Decoction | Stiff silkworm 10-15g, Cicada slough 5-10g, Rheum officinale(root and rhizome) 10-20g, Curcuma longa(rhizome) 10-15g, Astragalus mongholicus(root) 30-90g, Atractylodes macrocephala(rhizome) 10-30g, Poria 30-60g, Polyporus 10-20g, Alisma plantago-aquatica(tuber) 30g, Angelica sinensis(root) 10-15g, Lycopus lucidus 10-30g, Rhizoma Ligustici(chuan xiong) 5-10g, Aconitum carmichaelii(cooked sub-root) 10-20g | |
| Zhou 2014 | Tangshen-Shengqing-Jiangzhuo Granules | Stiff silkworm, Cicada slough, Rheum officinale(root and rhizome), Curcuma longa(rhizome), Astragalus mongholicus(root), Poria, Alisma plantago-aquatica(tuber), Paeonia suffruticosa(root bark), Plantago asiatica(seed), Cooked Rehmannia glutinosa(tuber), Dioscorea oppositifolia(tuber), Cornus officinalis(ripe fruit), Achyranthes bidentata(root), Angelica sinensis(root), Coptis chinensis(rhizome), Abelmoschus moschatus, Centella asiatica, Euonymus alatus(branches) | |
| Gao 2013 | Modified Shengjiang powder | Stiff silkworm 15g, Cicada slough 15g, Rheum officinale(root and rhizome) 9g , Curcuma longa(rhizome) 9g, Astragalus mongholicus(root) 60g, Angelica sinensis(root) 15g | |
| Liu 2012 | Modified Shengjiang powder | Stiff silkworm 10g, Cicada slough 15g, Rheum officinale(root and rhizome) 9g, Curcuma longa(rhizome) 6g, Astragalus mongholicus(root) 60g, Angelica sinensis(root) 15g, Poria 15g | |
| Tang 2011 | Modified Shengjiang powder | Stiff silkworm 10g, Cicada slough 15g, Rheum officinale(root and rhizome) 9g, Curcuma longa(rhizome) 6g, Astragalus mongholicus(root) 60g, Angelica sinensis(root) 15g, Poria 15g | |
| Li-2 2011 | Shengqing-Jiangzhuo Decoction | Stiff silkworm 12g, Cicada slough 6g, Rheum officinale(root and rhizome) 15g, Curcuma longa(rhizome) 6g, Astragalus mongholicus(root) 20g, Alisma plantago-aquatica(tuber) 15g, Cornus officinalis(ripe fruit) 15g, Rehmannia glutinosa(tuber) 24g, Dioscorea oppositifolia(tuber) 30g, Coptis chinensis(rhizome) 10g, Abelmoschus moschatus 30g, Centella asiatica 30g, Euonymus alatus(branches) 30g, Leech 10g | |
| Li-3 2011 | Buyang-Huanwu Decoction + Shengjiang powder | Stiff silkworm 12g, Cicada slough 12g, Rheum officinale(root and rhizome) 6g, Curcuma longa(rhizome) 12g, Astragalus mongholicus(root) 40g, Angelica sinensis(root) 15g, Rhizoma Ligustici(chuan xiong) 15g, Prunus persica(root) 15g, Lumbricus 15g, Carthamus tinctorius(flower) 10g, Prunus persica(seed) 10g | |
| Ji 2009 | Liuhuang-Tangshenkang Decoction | Stiff silkworm 6g, Cicada slough 6g, Rheum officinale(root and rhizome) 6g, Curcuma longa(rhizome) 12g, Astragalus mongholicus(root) 15g, Poria 15g, Alisma plantago-aquatica(tuber) 10g, Dioscorea oppositifolia(tuber) 15g, Rehmannia glutinosa(cooked tuber) 12g, Angelica sinensis(root) 10g, Paeonia suffruticosa(root bark) 12g, Prunus persica(root) 12g, Coptis chinensis(rhizome) 10g, Abelmoschus moschatus 15g, Centella asiatica 12g, Euonymus alatus(branches) 15g | |
| Li-1 2008 | Shengjiang-Guiling Decoction | Stiff silkworm 10g, Cicada slough 6g, Rheum officinale(root and rhizome) 10g, Curcuma longa(rhizome) 6g, Astragalus mongholicus(root) 15g, Poria 20g, Prunus persica(seed) 10g, Paeonia suffruticosa(root bark) 15g, Prunus persica(root) 16g, Cinnamomum cassia(twig) 10g | |

**Supplemental Table 4.** Summary of meta-analysis and subgroup analysis results.

| Outcomes  /subgroups | No. of studies | Heterogeneity | | Model | meta-analysis | |
| --- | --- | --- | --- | --- | --- | --- |
|  |  | P-value | I 2(%) |  | Effect size(95%CI ) | P-value |
| clinical efficacy | 18 | 0.33 | 11 | Random | RR=1.27(1.19,1.35) | P <0.001 |
| High risk | 2 | 0.28 | 14 | Random | RR=1.34(1.04,1.74) | 0.03 |
| Others | 17 | 0.29 | 15 | Random | RR=1.26(1.18,1.35) | P <0.001 |
| 24-h urinary protein | 14 | P <0.001 | 98 | Random | MD=-0.22(-0.27,-0.17) | P <0.001 |
| ＜3months | 7 | P <0.001 | 99 | Random | MD=-0.38(-0.51,-0.24) | P <0.001 |
| ≥3months | 6 | P <0.001 | 94 | Random | MD=-0.16(-0.27,-0.05) | P <0.001 |
| II/III | 4 | P <0.001 | 96 | Random | MD=-0.07(-0.11,-0.04) | P <0.001 |
| III/IV | 4 | 0.45 | 0 | Random | MD=-0.10(-0.13,-0.08) | P <0.001 |
| IV | 5 | P <0.001 | 99 | Random | MD=-0.67(-1.11,-0.22) | P <0.01 |
| looser | 3 | 0.01 | 77 | Random | MD=-0.09(-0.17,-0.01) | P <0.05 |
| stricter | 4 | P <0.001 | 99 | Random | MD=-0.55(-0.84,-0.26) | P <0.001 |
| lower | 4 | P <0.001 | 99 | Random | MD=-0.65(-1.16,-0.13) | 0.01 |
| higher | 6 | P <0.001 | 95 | Random | MD=-0.17(-0.26,-0.07) | 0.001 |
| Non-mentioned | 5 | P <0.001 | 89 | Random | MD=-0.09(-0.15,-0.03) | P =0.003 |
| Mentioned | 9 | P <0.001 | 98 | Random | MD=-0.39(-0.54,-0.24) | P <0.001 |
| A | 1 | - | - | - | MD=-0.28(-0.78,0.22) | - |
| B | 3 | P <0.001 | 98 | Random | MD=-0.25(-0.50,0.00) | 0.05 |
| C | 0 | - | - | - | - | - |
| D | 10 | P <0.001 | 98 | Random | MD=-0.29(-0.40,-0.19) | P <0.001 |
| High risk | 2 | P <0.001 | 95 | Random | MD=-0.11(-0.26,0.03) | 0.12 |
| Others | 12 | P <0.001 | 98 | Random | MD=-0.25(-0.31,-0.19) | P <0.001 |
| UAER | 6 | P <0.001 | 85 | Random | MD=-40.26(-52.88,-27.64) | P <0.001 |
| ＜2months | 2 | 0.15 | 52 | Random | MD=-50.72(-58.87,-42.58) | P <0.001 |
| ≥2months | 4 | 0.19 | 37 | Random | MD=-29.14(-40.14,-18.14) | P <0.001 |
| High risk | 0 | - | - | - | - | - |
| Others | 6 | P <0.001 | 85 | Random | MD=-40.26(-52.88,-27.64) | P <0.001 |
| mALB | 6 | P <0.001 | 82 | Random | MD=-15.23(-25.17,-5.29) | P <0.01 |
| Non-mentioned | 4 | 0.45 | 0 | Random | MD=-20.87(-29.06,-12.69) | P <0.001 |
| Mentioned | 2 | P <0.001 | 95 | Random | MD=-6.80(-24.36,10.75) | 0.45 |
| A | 0 | - | - | - | - | - |
| B | 1 | - | - | - | MD=-15.55(-19.69,-11.41) | - |
| C | 0 | - | - | - | - | - |
| D | 5 | P <0.001 | 81 | Random | MD=-16.11(-30.35,-1.88) | 0.03 |
| High risk | 2 | 0.003 | 89 | Random | MD=-14.26(-50.09,-21.56) | 0.44 |
| Others | 4 | 0.65 | 0 | Random | MD=-16.17(-19.91,-12.43) | P <0.001 |
| BUN | 13 | P <0.001 | 98 | Random | MD=-1.09(-1.64,-0.54) | P <0.001 |
| ＜3months | 7 | P <0.001 | 99 | Random | MD=-1.32(-1.96,-0.68) | P <0.001 |
| ≥3months | 5 | 0.04 | 59 | Random | MD=-0.56(-0.98,-0.15) | P <0.01 |
| II/III | 3 | P <0.001 | 95 | Random | MD=-1.06(-2.07,-0.05) | P <0.05 |
| III/IV | 3 | 0.02 | 75 | Random | MD=-0.54(-1.39,0.32) | 0.22 |
| IV | 6 | P <0.001 | 99 | Random | MD=-1.29(-2.04,-0.53) | P <0.001 |
| lower | 4 | 0.001 | 81 | Random | MD=-0.35(-0.98,0.29) | 0.28 |
| higher | 4 | 0.08 | 56 | Random | MD=-1.23(-1.76,-0.70) | P <0.001 |
| Non-mentioned | 2 | 0.19 | 42 | Random | MD=-0.49(-1.55,0.57) | 0.36 |
| Mentioned | 11 | P <0.001 | 99 | Random | MD=-1.17(-1.76,-0.58) | P <0.001 |
| A | 1 | - | - | - | MD=0.21(-1.55,1.97) | - |
| B | 4 | P <0.001 | 93 | Random | MD=-0.70(-1.24,-0.15) | 0.01 |
| C | - | - | - | - | - | - |
| D | 8 | P <0.001 | 95 | Random | MD=-1.34(-1.75,-0.93) | P <0.001 |
| High risk | 1 | - | - | - | MD=-0.14(-21.09,-20.81) | - |
| Others | 10 | P <0.001 | 99 | Random | MD=-1.17(-1.76,-0.58) | P <0.001 |
| Scr | 12 | P <0.001 | 91 | Random | MD=-9.87(-13.48,-6.27) | P <0.001 |
| ＜3months | 6 | P <0.001 | 90 | Random | MD=-9.76(-14.74,-4.78) | P <0.001 |
| ≥3months | 5 | 0.83 | 0 | Random | MD=-4.27(-5.83,-2.72) | P <0.001 |
| II/III | 2 | 0.001 | 91 | Random | MD=-3.34(-12.41,5.72) | 0.47 |
| III/IV | 4 | P <0.01 | 80 | Random | MD=-14.63(-27.26,-2.00) | P <0.05 |
| IV | 5 | P <0.001 | 86 | Random | MD=-8.11(-11.52,-4.71) | P <0.001 |
| lower(Scr) | 9 | P <0.001 | 75 | Random | MD=-5.26(-7.78,-2.75) | P <0.001 |
| higher(Scr) | 3 | P <0.001 | 90 | Random | MD=-19.94(-30.41,-9.47) | P <0.001 |
| Lower(FBG) | 4 | 0.10 | 52 | Random | MD=-3.29(-8.14,1.56) | 0.18 |
| Higher(FBG) | 3 | P <0.001 | 96 | Random | MD=-13.50(-30.63,3.63) | 0.12 |
| Non-mentioned | 3 | P <0.001 | 87 | Random | MD=-14.97(-33.97,4.04) | 0.12 |
| Mentioned | 9 | P <0.001 | 93 | Random | MD=-9.99(-13.91,-6.07) | P <0.001 |
| A | 1 | - | - | - | MD=-13.70(-48.69,21.29) | - |
| B | 3 | 0.005 | 81 | Random | MD=-3.75(-7.87,0.38) | 0.08 |
| C | - | - | - | - | - | - |
| D | 8 | P <0.001 | 93 | Random | MD=-13.73(-20.21,-7.26) | P <0.001 |
| High risk | 0 | - | - | - | - | - |
| Others | 12 | P <0.001 | 91 | Random | MD=-9.87(-13.48,-6.27) | P <0.001 |
| FBG | 14 | P <0.001 | 80 | Random | MD=-0.78(-1.09,-0.48) | P <0.001 |
| ＜3months | 6 | P <0.001 | 86 | Random | MD=-0.82(-1.29,-0.36) | P <0.001 |
| ≥3months | 7 | 0.03 | 56 | Random | MD=-0.66(-1.11,-0.20) | P <0.01 |
| II/III | 5 | P <0.001 | 81 | Random | MD=-0.97(-1.41,-0.54) | P <0.001 |
| III/IV | 4 | 0.26 | 26 | Random | MD=-1.04(-1.57,-0.51) | P <0.001 |
| IV | 3 | P <0.001 | 86 | Random | MD=-0.10(-1.32,1.12) | 0.87 |
| Non-mentioned | 5 | 0.11 | 47 | Random | MD=-0.75(-1.18,-0.31) | P <0.001 |
| Mentioned | 9 | P <0.001 | 83 | Random | MD=-0.79(-1.17,-0.41) | P <0.001 |
| A | 1 | - | - | - | MD=-1.10(-2.02,-0.18) | - |
| B | 2 | 0.65 | 0 | Random | MD=-1.36(-1.52,-1.20) | P <0.001 |
| C | 1 | - | - | - | MD=-0.02(-0.56,0.52) | - |
| D | 10 | P <0.001 | 68 | Random | MD=-0.67(-1.03,-0.03) | P <0.001 |
| High risk | 3 | 0.003 | 82 | Random | MD=-0.04(-1.64,1.72) | 0.97 |
| Others | 11 | P <0.001 | 74 | Random | MD=-0.93(-1.21,-0.65) | P <0.001 |
| 2hPG | 7 | P <0.001 | 80 | Random | MD=-1.25(-2.10,-0.40) | P <0.01 |
| ＜3months | 4 | P <0.001 | 86 | Random | MD=-1.60(-2.94,-0.27) | P <0.05 |
| ≥3months | 3 | 0.02 | 76 | Random | MD=-0.84(-2.29,0.61) | 0.26 |
| II/III | 4 | P <0.001 | 86 | Random | MD=-1.00(-2.23,0.24) | 0.11 |
| III/IV | 1 | - | - | - | MD=-1.19(-2.28,-0.10) | - |
| IV | 2 | 0.94 | 0 | Random | MD=-1.97(-3.00,-0.93) | P <0.001 |
| Non-mentioned | 4 | 0.03 | 66 | Random | MD=-0.93(-1.97,0.11) | 0.08 |
| Mentioned | 3 | P <0.001 | 90 | Random | MD=-1.78(-3.70,0.15) | 0.07 |
| A | - | - | - | - | - | - |
| B | - | - | - | - | - | - |
| C | - | - | - | - | - | - |
| D | 7 | P <0.001 | 80 | Random | MD=-1.25(-2.10,-0.40) | 0.004 |
| High risk | 3 | 0.08 | 60 | Random | MD=-2.12(-3.34,-0.90) | P <0.001 |
| Excluded | 4 | 0.02 | 68 | Random | MD=-0.63(-1.45,-0.19) | 0.13 |
| HbA1c | 12 | P <0.001 | 75 | Random | MD=-0.48(-0.69,-0.26) | P <0.001 |
| ＜3months | 6 | 0.001 | 75 | Random | MD=-0.55(-0.84,-0.27) | P <0.001 |
| ≥3months | 6 | P <0.001 | 77 | Random | MD=-0.37(-0.80,0.05) | 0.08 |
| II/III | 5 | 0.09 | 50 | Random | MD=-0.38(-0.61,-0.16) | 0.001 |
| III/IV | 3 | P <0.001 | 89 | Random | MD=-0.46(-1.47,0.56) | 0.38 |
| IV | 3 | 0.03 | 72 | Random | MD=-0.56(-1.02,-0.10) | P <0.05 |
| Non-mentioned | 5 | 0.004 | 74 | Random | MD=-0.55(-0.98,-0.13) | P <0.05 |
| Mentioned | 7 | P <0.001 | 75 | Random | MD=-0.43(-0.71,-0.16) | P <0.01 |
| A | 1 | - | - | - | MD=-0.50(-0.16,1.16) | - |
| B | 2 | 0.06 | 73 | Random | MD=-0.37(-0.74,-0.01) | 0.05 |
| C | 1 | - | - | - | MD=-0.52(-0.95,-0.09) | - |
| D | 8 | 0.008 | 63 | Random | MD=-0.60(-0.88,-0.32) | P <0.001 |
| High risk | 3 | 0.86 | 0 | Random | MD=-0.57(-0.88,-0.26) | P <0.001 |
| Others | 9 | P <0.001 | 80 | Random | MD=-0.45(-0.71,-0.19) | P <0.001 |
| TC | 7 | 0.63 | 0 | Random | MD=-0.74(-0.88,-0.61) | P <0.001 |
| ＜3months | 4 | 0.59 | 0 | Random | MD=-0.70(-0.90,-0.51) | P <0.001 |
| ≥3months | 2 | 0.61 | 0 | Random | MD=-0.61(-0.93,-0.29) | P <0.001 |
| II/III | 3 | 0.80 | 0 | Random | MD=-0.59(-0.86,-0.32) | P <0.001 |
| III/IV | 1 | - | - | - | MD=-0.68(-1.09,-0.27) | - |
| IV | 2 | 0.29 | 12 | Random | MD=-0.75(-1.02,-0.47) | P <0.001 |
| looser | 3 | 0.77 | 0 | Random | MD=-0.60(-0.85,-0.36) | P <0.001 |
| stricter | 3 | 0.55 | 0 | Random | MD=-0.74(-0.97,-0.52) | P <0.001 |
| lower | 2 | 0.52 | 0 | Random | MD=-0.58(-0.86,-0.31) | P <0.001 |
| higher | 4 | 0.58 | 0 | Random | MD=-0.81(-0.97,-0.64) | P <0.001 |
| Non-mentioned | 2 | 0.61 | 0 | Random | MD=-0.61(-0.93,-0.29) | P <0.001 |
| Mentioned | 5 | 0.51 | 0 | Random | MD=-0.77(-0.93,-0.62) | P <0.001 |
| A | 0 | - | - | - | - | - |
| B | 2 | 0.60 | 0 | Random | MD=-0.56(-0.87,-0.24) | P <0.001 |
| C | 0 | - | - | - | - | - |
| D | 5 | 0.68 | 0 | Random | MD=-0.79(-0.94,-0.64) | P <0.001 |
| High risk | 2 | 0.62 | 0 | Random | MD=-0.79(-1.04,-0.55) | P <0.001 |
| Others | 5 | 0.43 | 0 | Random | MD=-0.72(-0.89,-0.56) | P <0.001 |
| TG | 6 | P <0.001 | 90 | Random | MD=-0.54(-0.82,-0.25) | P <0.001 |
| ＜3months | 3 | P <0.001 | 96 | Random | MD=-0.63(-1.20,-0.06) | P <0.05 |
| ≥3months | 2 | 0.73 | 0 | Random | MD=-0.41(-0.65,-0.17) | P <0.001 |
| II/III | 2 | 0.01 | 84 | Random | MD=-0.33(-0.67,0.02) | 0.06 |
| III/IV | 1 | - | - | - | MD=-0.49(-0.99,0.01) | - |
| IV | 2 | P <0.001 | 96 | Random | MD=-0.83(-1.69,0.03) | 0.06 |
| looser | 3 | 0.04 | 68 | Random | MD=-0.36(-0.62,-0.09) | P <0.001 |
| stricter | 2 | P <0.001 | 96 | Random | MD=-0.88(-1.69,0.03) | 0.06 |
| lower | 2 | 1.00 | 0 | Random | MD=-0.49(-0.63,-0.35) | P <0.001 |
| higher | 4 | P <0.001 | 94 | Random | MD=-0.56(-1.00,-0.11) | 0.01 |
| Non-mentioned | 2 | 0.12 | 0 | Random | MD=-0.41(-0.65,-0.17) | P <0.001 |
| Mentioned | 4 | P <0.001 | 94 | Random | MD=-0.58(-0.95,-0.20) | P <0.001 |
| A | 0 | - | - | - | - | - |
| B | 1 | - | - | - | MD=-0.49(-0.64,-0.34) | - |
| C | 0 | - | - | - | - | - |
| D | 5 | P <0.001 | 92 | Random | MD=-0.55(-0.93,-0.16) | 0.006 |
| High risk | 2 | P <0.001 | 98 | Random | MD=-0.70(-1.81,0.40) | 0.21 |
| Others | 4 | 0.91 | 0 | Random | MD=-0.45(-0.55,-0.35) | P <0.001 |
| Adverse events | 13 | 0.92 | 0 | Random | RR=1.41(0.42,4.67) | 0.58 |
| High risk | 4 | - | - | - | RR=3.00(0.13,70.83) | 0.50 |
| Others | 9 | 0.89 | 0 | Random | RR=1.24(0.34,4.53) | 0.75 |

Abbreviations: UAER, Urinary Albumin Excretion Rate; mALB, Microalbuminuria; BUN, Blood Urea Nitrogen; Scr, Serum Creatinine; FBG, Fasting Blood Glucose; 2hPG, 2-h post-load Plasma Glucose; HbA1c, Hemoglobin A1c; TC, Total Cholesterol; TG, Triglycerides.

**Supplemental Table 5.** Egger’s test to assess publication bias.

| Std_Eff | Coef. | Std.Err. | t | P>\|t\| | [95% Conf. Interval] | |
| --- | --- | --- | --- | --- | --- | --- |
| Publication bias of clinical efficacy | 2.734709 | .3878451 | 7.05 | 0.000 | 1.912514 | 3.556904 |
| Publication bias of 24-h urinary protein | 10.85212 | 2.855586 | 3.80 | 0.003 | 4.630335 | 17.07391 |
| Publication bias of BUN | 15.03928 | 2.200584 | 6.83 | 0.000 | 10.19583 | 19.88274 |
| Publication bias of Scr | 18.20393 | 4.259268 | 4.27 | 0.002 | 8.713689 | 27.69417 |
| Publication bias of FBG | 12.14298 | 5.714501 | 2.12 | 0.055 | -.3078523 | 24.59381 |
| Publication bias of HbA1c | 8.088335 | 4.791418 | 1.69 | 0.122 | -2.58761 | 18.76428 |

Abbreviations: BUN, Blood Urea Nitrogen; Scr, Serum Creatinine; FBG, Fasting Blood Glucose; HbA1c, Hemoglobin A1c.

**Supplemental Table 6.** Results of Trim and filling method.

| Outcome | Method | Pooled Est | 95%CI | | Asymptotic | | No. of studies |
| --- | --- | --- | --- | --- | --- | --- | --- |
|  |  |  | Lower | Upper | z_value | p_value |  |
| Clinical efficacy | Random | 1.222 | 1.145 | 1.305 | 5.987 | 0.000 | 24 |
| 24-h urinary protein | Random | 3.683 | 2.397 | 5.659 | 5.948 | 0.000 | 14 |
| BUN | Random | 5.999 | 2.842 | 12.663 | 4.700 | 0.000 | 13 |
| Scr | Random | 2.697 | 1.674 | 4.345 | 4.079 | 0.000 | 12 |

Abbreviation: BUN, Blood Urea Nitrogen; Scr, Serum Creatinine.

**Supplemental Table 7.** Overall GRADE profile.

| No. of studies | Certainty assessment | | | | | No. of patients (E/C) | Effect (95%CI) | Overall certainty of evidence |
| --- | --- | --- | --- | --- | --- | --- | --- | --- |
|  | Risk of bias | Inconsistency | Indirectness | Imprecision | Publication bias |  |  |  |
| Clinical efficacy | | | | | | | | |
| 19 | Serious^a^ | Not serious | Not serious | Not serious | Strongly suspected^b^ | 673/658 | RR=1.27(1.19,1.35) | ⨁⨁◯◯ |
|  |  |  |  |  |  |  |  | Low |
| 24-h urinary protein | | | | | | | | |
| 14 | Serious^a^ | Not serious | Not serious | Not serious | Strongly suspected^b^ | 504/493 | MD=-0.22(-0.27,-0.17) | ⨁⨁◯◯ |
|  |  |  |  |  |  |  |  | Low |
| UAER | | | | | | | | |
| 7 | Serious^a^ | Not serious | Not serious | Not serious | None | 254/256 | MD=-40.26(-52.88,-27.64) | ⨁⨁⨁◯ |
|  |  |  |  |  |  |  |  | Moderate |
| mALB | | | | | | | | |
| 6 | Serious^c^ | Not serious | Not serious | Not serious | None | 206/207 | MD=-15.23(-25.17,-5.29) | ⨁⨁⨁◯ |
|  |  |  |  |  |  |  |  | Moderate |
| BUN | | | | | | | | |
| 14 | Serious^a^ | Not serious | Not serious | Not serious | Strongly suspected^b^ | 517/501 | MD=-1.09(-1.64,-0.54) | ⨁⨁◯◯ |
|  |  |  |  |  |  |  |  | Low |
| Scr | | | | | | | | |
| 13 | Serious^a^ | Not serious | Not serious | Not serious | Strongly suspected^b^ | 477/461 | MD=-9.87(-13.48,-6.27) | ⨁⨁◯◯ |
|  |  |  |  |  |  |  |  | Low |
| FBG | | | | | | | | |
| 15 | Serious^a^ | Not serious | Not serious | Not serious | None | 505/487 | MD=-0.78(-1.09,-0.48) | ⨁⨁⨁◯ |
|  |  |  |  |  |  |  |  | Moderate |
| 2hPG | | | | | | | | |
| 7 | Serious^a^ | Not serious | Not serious | Not serious | None | 230/229 | MD=-1.25(-2.10,-0.40) | ⨁⨁⨁◯ |
|  |  |  |  |  |  |  |  | Moderate |
| HbA1c | | | | | | | | |
| 12 | Serious^a^ | Not serious | Not serious | Not serious | None | 409/410 | MD=-0.48(-0.69,-0.26) | ⨁⨁⨁◯ |
|  |  |  |  |  |  |  |  | Moderate |
| TC | | | | | | | | |
| 7 | Serious^a^ | Not serious | Not serious | Not serious | None | 259/254 | MD=-0.74(-0.88,-0.61) | ⨁⨁⨁◯ |
|  |  |  |  |  |  |  |  | Moderate |
| TG | | | | | | | | |
| 6 | Serious^a^ | Not serious | Not serious | Not serious | None | 218/211 | MD=-0.54(-0.82,-0.25) | ⨁⨁⨁◯ |
|  |  |  |  |  |  |  |  | Moderate |
| Adverse events | | | | | | | | |
| 13 | Serious^a^ | Not serious | Not serious | Seriousd | None | 391/392 | RR=1.41(0.42,4.67) | ⨁⨁◯◯ |
|  |  |  |  |  |  |  |  | Low |

Abbreviations: E, Experimental group; C, Control group; UAER, Urinary Albumin Excretion Rate; mALB, Microalbuminuria; BUN, Blood Urea Nitrogen; Scr, Serum Creatinine; FBG, Fasting Blood Glucose; 2hPG, 2-h post-load Plasma Glucose; HbA1c, Hemoglobin A1c; TC, Total cholesterol; TG, Triglycerides.

Explanations: a.Several studies used inappropriate randomizations. b.Egger's test showed publication bias. c.Several studies' randomizations were unprovided. d.Few events were observed and follow-ups were insufficient.
