# Supplementary material for: Efficacy and safety of the traditional Chinese formula Shengjiang powder combined with conventional therapy in the treatment of diabetic kidney disease: a systematic review and meta-analysis
Source: Front Endocrinol (Lausanne). 2024 Jul 23;15:1400939. doi: 10.3389/fendo.2024.1400939 (PMC11300290; doi:10.3389/fendo.2024.1400939)
Supplement: Supplementary file 2 [file DataSheet_2.docx]

Supplementary Material

# Supplementary Figures





**Supplementary Figure 1.** Forest plots of Subgroup analyses (1). Forest plots of (A) 24-h urinary protein subgroups based on duration, (B) 24-h urinary protein subgroups based on baseline FBG levels, (C) BUN subgroups based on duration, (D) BUN subgroups based on blood glucose control levels, (E) Scr subgroups based on duration, and (F) FBG subgroups based on duration. MDs among subgroups had no statistical difference, respectively. Abbreviations: FBG, Fasting Blood Glucose; BUN, Blood Urea Nitrogen; Scr, Serum Creatinine.


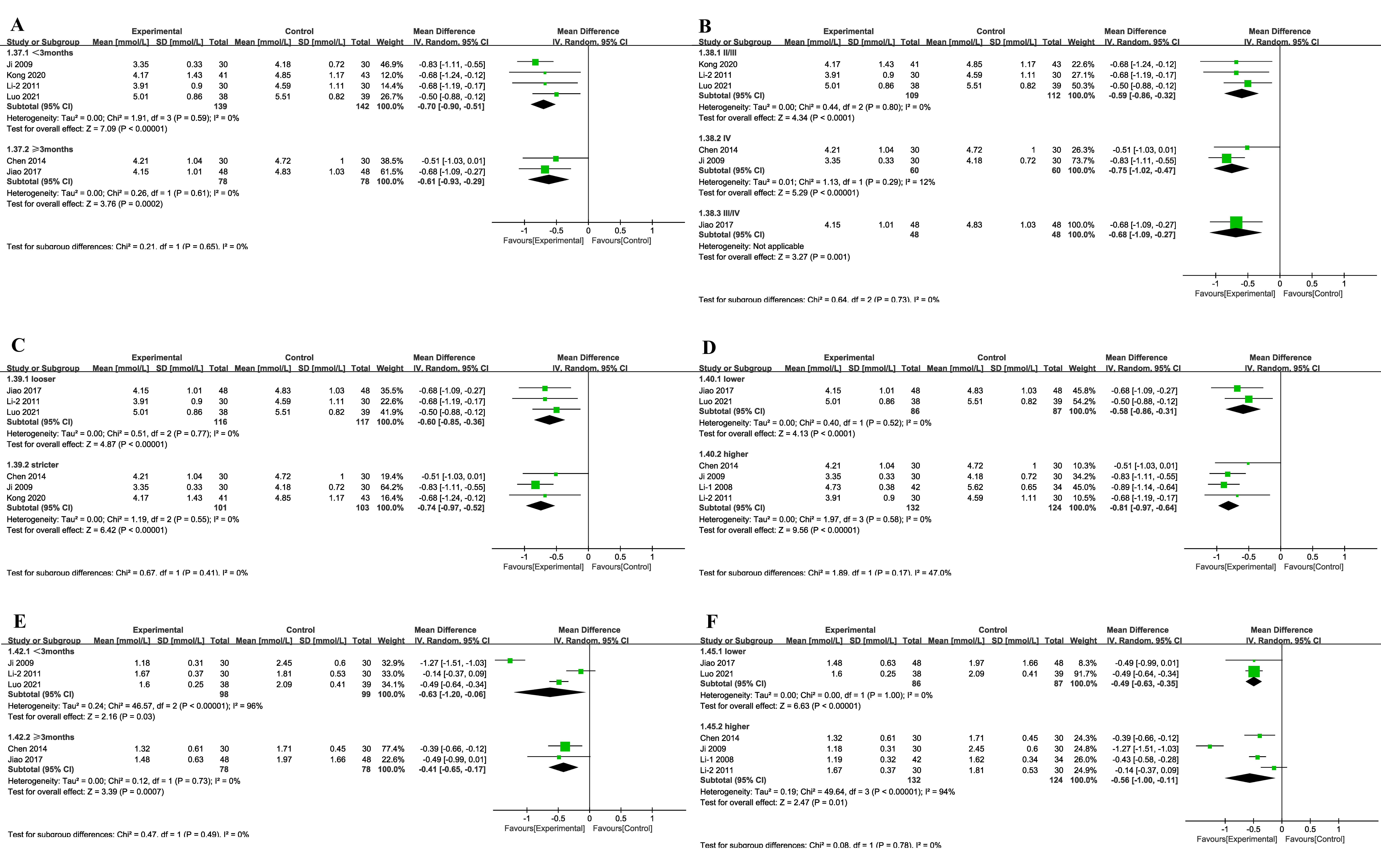


**Supplementary Figure 2.** Forest plots of Subgroup analyses (2). Forest plots of (A) TC subgroups based on duration, (B) TC subgroups based on stage, (C) TC subgroups based on blood glucose control levels, (D) TC subgroups based on baseline FBG levels, (E) TG subgroups based on duration, and (F) TG subgroups based on baseline FBG levels. MDs among subgroups had no statistical difference, respectively. Abbreviations: FBG, Fasting Blood Glucose; TC, Total cholesterol; TG, Triglycerides.





**Supplementary Figure 3.** Funnel plots. Funnel plots of (A)clinical efficacy, (B)24-h urinary protein, (C)BUN, (D)Scr, (E)FBG, and (F)HbA1c. Partial asymmetry existed between the two sides of funnel plots, indicating that there may be publication bias and small sample effects. Abbreviations: BUN, Blood Urea Nitrogen; Scr, Serum Creatinine; FBG, Fasting Blood Glucose; HbA1c, Hemoglobin A1c.


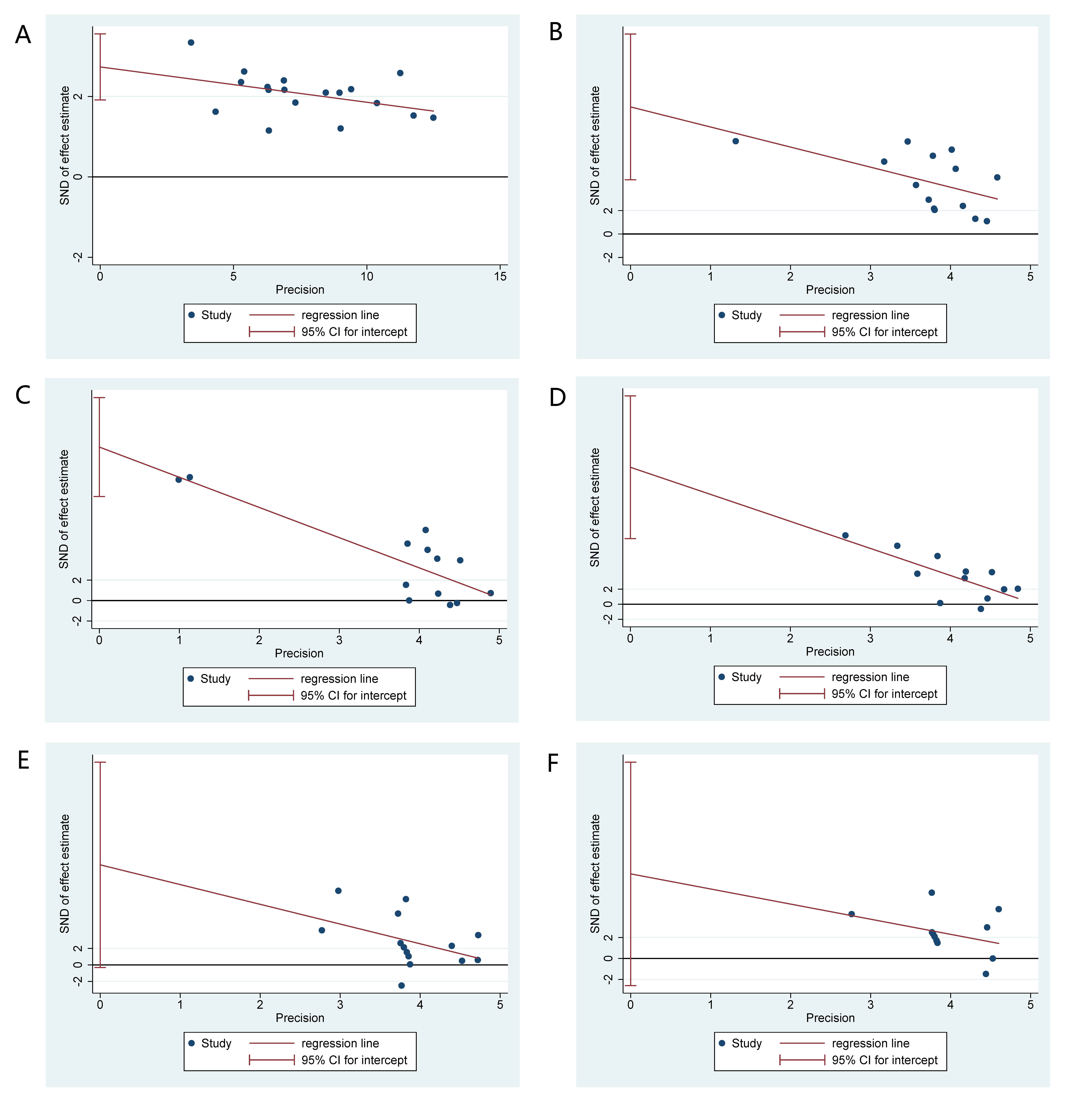


**Supplementary Figure 4.** Publication bias tests. Publication bias test of (A)clinical efficacy, (B)24-h urinary protein, (C)BUN, (D)Scr, (E)FBG, (F)HbA1c. Egger’s test detected that clinical efficacy, 24-h urinary protein, and FBG may indicate publication bias in their evaluated studies (P ＜ 0.05). Abbreviations: BUN, Blood Urea Nitrogen; Scr, Serum Creatinine; FBG, Fasting Blood Glucose; HbA1c, Hemoglobin A1c.


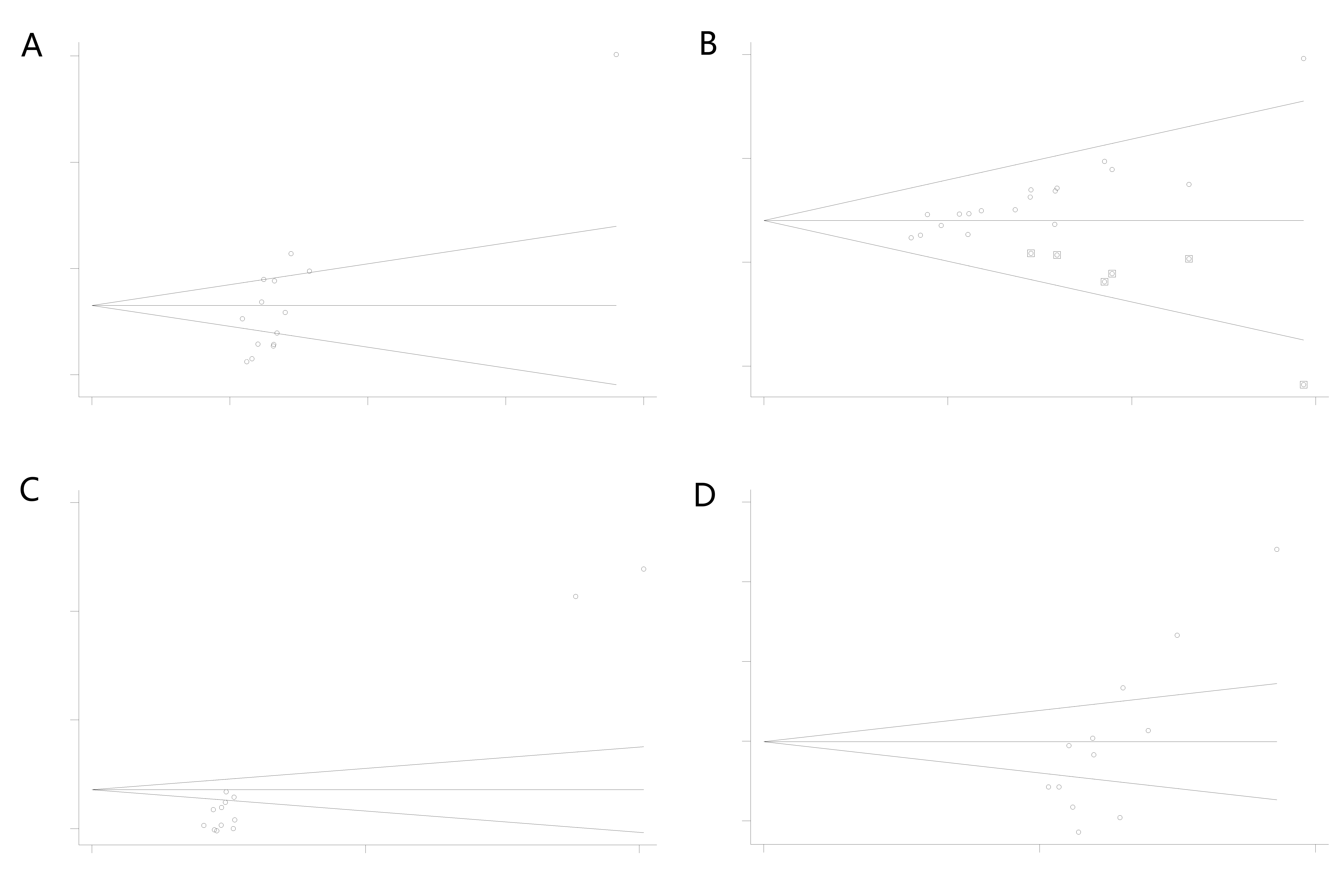


**Supplementary Figure 5.** Trim and filling method results of (A)clinical efficacy, (B)24-h urinary protein, (C)BUN, and (D)Scr. The statistical significance still existed in 24-h urinary protein before and after trimming and filling.Besides, the clinical efficacy, BUN, and Scr were not filled after linear iteration. It showed publication bias having no significant effect on these outcomes, and that results were stable. Abbreviations: BUN, Blood Urea Nitrogen; Scr, Serum Creatinine.





**Supplementary Figure 6.** Forest plots of subgroups based on whether mentioned the specific dose. Forest plots of (A) 24-h urinary protein, (B) mALB, (C) BUN, (D) Scr, (E) FBG, (F) 2hPG, (G) HbA1c, (H) TC, and (I) TG subgroups. Abbreviations: mALB, Microalbuminuria; BUN, Blood Urea Nitrogen; Scr, Serum Creatinine; FBG, Fasting Blood Glucose; 2hPG, 2-h post-load Plasma Glucose; HbA1c, Hemoglobin A1c; TC, Total Cholesterol; TG, Triglycerides.





**Supplementary Figure 7.** Forest plots of subgroups based on differences in specific diagnostic criteria. Forest plots of (A) 24-h urinary protein, (B) mALB, (C) BUN, (D) Scr, (E) FBG, (F) 2hPG, (G) HbA1c, (H) TC, and (I) TG subgroups. Abbreviations: mALB, Microalbuminuria; BUN, Blood Urea Nitrogen; Scr, Serum Creatinine; FBG, Fasting Blood Glucose; 2hPG, 2-h post-load Plasma Glucose; HbA1c, Hemoglobin A1c; TC, Total Cholesterol; TG, Triglycerides.


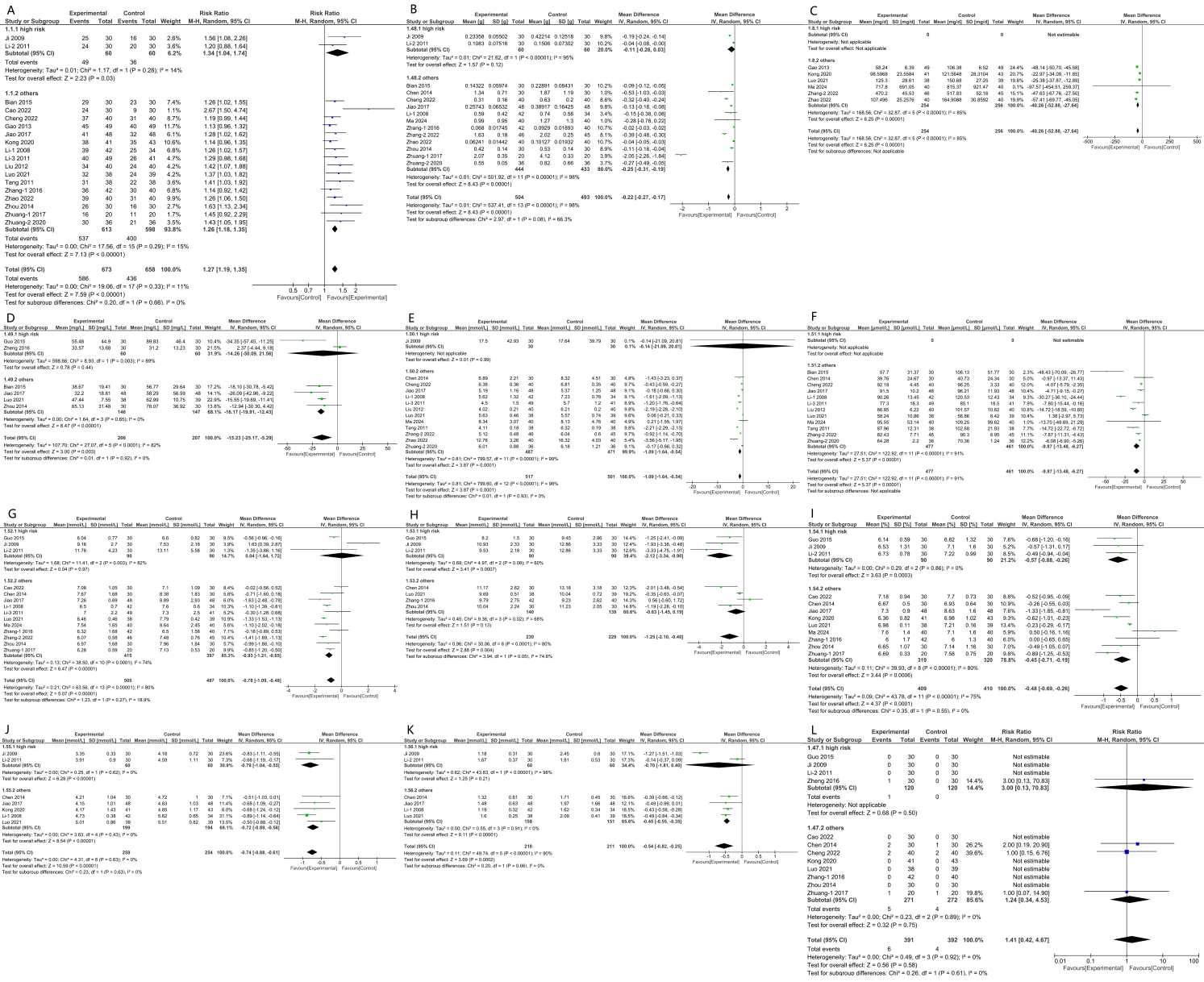


**Supplementary Figure 8.** Forest plots of subgroups based on the risk of bias. Forest plots of (A) clinical efficacy, (B) 24-h urinary protein, (C) UAER; (D)mALB, (E) BUN, (F) Scr, (G) FBG, (H) 2hPG, (I) HbA1c, (J) TC, (K) TG, and (L) adverse events subgroups. Abbreviations: UAER, Urinary Albumin Excretion Rate; mALB, Microalbuminuria; BUN, Blood Urea Nitrogen; Scr, Serum Creatinine; FBG, Fasting Blood Glucose; 2hPG, 2-h post-load Plasma Glucose; HbA1c, Hemoglobin A1c; TC, Total Cholesterol; TG, Triglycerides.
